# Spore-based innovative paper-strip biosensor for the rapid detection of ß-lactam group in milk

**DOI:** 10.1038/s41598-022-26466-7

**Published:** 2022-12-19

**Authors:** Prashant Goel, Raghu Hirikyathanahalli Vishweswaraiah, Naresh Kumar

**Affiliations:** Dairy Microbiology Division, ICAR-National Dairy Research Institute, Karnal, India

**Keywords:** Biological techniques, Microbiology, Health care

## Abstract

The study's goal was to develop a spore-based paper strip biosensor for detecting ß-lactam antibiotics in milk using the enzyme induction principle. A new spore-based paper strip biosensor has been developed after important operating parameters such as spore volume, substrate volume, exposure time and temperature, and incubation time and temperature were optimised. The limit of detection for various ß-lactam antibiotics, including amoxicillin, penicillin, ampicillin, carbenicillin, cloxacillin, nafcillin, oxacillin, cephalothin, cefalexin, cefoxitin, cefazolin, and cefuroxime, was determined in milk with detection sensitivity of 1 ppb, 2 ppb, 2 ppb, 10 ppb, 10 ppb, 10 ppb, 20 ppb, 10 ppb 1000 ppb, 10 ppb 300 ppb and 100 ppb, respectively. It was also tested with other contaminants such non-ß-lactam antibiotics, pesticides, aflatoxin, heavy metals, and other chemical contaminants, and no interference was found, indicating that the created biosensor had a low rate of false positive and negative results. In comparison to the AOAC-approved CHARM-ROSA ß-lactam strip test, which identified 7 raw milk and zero pasteurised milk samples positive for ß-lactam antibiotics, the sensor was further analysed and verified using 200 raw milk and 105 pasteurised milk samples. This indicates a perfect match between our biosensor and the AOAC-approved CHARM-ROSA ß-lactam strip test. The developed spore-based paper strip biosensors are expected to be useful in the rapid and cost-effective detection of ß-lactam antibiotic residues in milk samples at the dairy farm, reception dock, and production units, respectively.

## Introduction

Paper based biosensors have become the backbone of the diagnostic industry. In recent years, paper has been employed as a platform for creating devices that detect certain analytes using functionalized sensors^1,2^. The notable work on enzyme electrodes by Clark and Lyons in 1962 denoted the beginning of the field of biosensors^3^. A biosensor is a device that combines a bio-recognition molecule with a transducer and transforms the recognition event into an analytical signal^4^. They have been used in a wide range of applications, including medical diagnostics, food quality assurance, environmental monitoring, and industrial process control, as well as the detection of biological agents^5^. Paper based biosensors have been intensively researched for building point-of-care (POC) devices and gaining popularity in diagnostics and pollution monitoring because they are biodegradable, biocompatible, and need less equipment to manufacture^6^. However, the innovation in biosensors is quite a challenging task in relations of its cost and shelf-stability of bio-recognition molecule.

In today's world, many biological molecules are engaged in the sensing and recognition processes. These include cells, nucleic acids, enzymes, antibodies, proteins, peptides, etc.^7^. Many biological recognition molecules are existing naturally and based on their sensitivity and specificity, they can be used for the recognition of various components. Our research group at ICAR-NDRI used bacterial spores as a sensing molecule to detect antibiotics, pesticides and heavy metals in milk^8–12^.

Till date, the biphasic transformation of spores from dormant to vegetative cell type and vice versa has been employed as the working principle for spore-based sensors. These sensors use a variety of biomarkers, including dipicolinic acid (DPA) and various enzymes produced during spore germination. The presence of a specific analyte (contaminant) can indicate a change in the germination process, which can be detected using any of the aforementioned germination biomarkers. Spores of several bacillus species have been employed as bio-sensing elements in the development of biosensors to detect a variety of biological and chemical pollutants^13^.

Antibiotics have long been used in conventional livestock production for treating disease, preventing disease, enhancing feed efficiency, increase growth and productivity in animals and animal products^14^. Antibiotics given to dairy animals do enter the milk to some extent, and each antibiotic has a withdrawal (waiting) period during which the drug's concentration in the animal tissues decreases and is eliminated from the body. Antibiotics are the most regularly used antimicrobial drugs in the fight against mastitis-causing bacteria. The presence of ß-lactam antibiotics in the food chain is a major source of concern, as they can impede the expansion of starter cultures used in the dairy sector, cause allergic reactions in sensitive individuals, and modify the flora of the intestines^15^. The World Health Organization has classified the majority of these antibiotics as "critically important"^16^. Recently, FSSAI has specified the Maximum Residue Limits (MRLs) for these contaminants in milk with test methods involving conventional techniques like LC–MS, HPLC which requires complex sample processing steps, analytical procedure and skilled manpower for operation and therefore, availability of cost effective ready to use tests would be of great help to industry in regulatory compliance of these contaminants in dairy foods^17^.

In recent past, Paper Based Analytical Devices (PADs) have become popular because their fabrication is simple, low cost, portable, response time is fast, passive liquid transport and disposable in nature^18^. Our research group at ICAR-NDRI has developed rapid, novel spore based concepts /assays for detection of antibiotics^19,20^ and pesticides in milk^8–11^. DPA based kit is working on spore germination inhibition principle and detection of antimicrobial residues is based on development of yellow colour^20,21^. This test was further, transformed on paper strip for detection of antimicrobials based on release of marker enzyme after spore germination and development of blue colour on strip was indicative of absence of antimicrobials when incubated for 60 min at 64 °C^22^. The developed concepts are superior over existing prior-art in terms of sensitivity, selectivity, shelf-stability, cost per test, portable nature and requires minimal setup for testing. The concepts were validated from NABL accredited labs and technologies were commercialised to various entrepreneurs in India. In our lab, a chromogenic substrate based assay for specific detection of ß-lactam antibiotics in milk have also been developed based on substrate competition principle^23^, however, the test needs to be transformed on paper strip.

Current research describes a spore-based paper strip biosensor that uses the induction principle to detect the ß-lactam group of antibiotics in milk. The presence of ß-lactam antibiotics in milk poses a number of health hazards, including allergies, cancer, and microbial drug resistance. Antibiotics in milk must be detected using simple, low-cost technologies, particularly in remote locations where medical services are few. The device's design, manufacture, and operation are shown here, as well as the detection of ß-lactam antibiotics in milk samples. Finally, the device will aid in the prevention of pathogenic microorganisms developing antimicrobial resistance (AMR) (Figure S1–S15).

## Material and methods

### Chemicals and reagents

The propagation medium was prepared by following the protocol in our recent work^24^. The stock solution of the chromogenic substrate nitrocefin was prepared in 500 µL organic solvent with the addition of 5 mg nitrocefin (Sigma Aldrich, U.S.A; HiMedia, Mumbai, India). ß-lactam antibiotics (HiMedia) solution was prepared by diluting standard stock solution in milk and stored at − 20 °C. Potassium phosphate buffer (Hi-Media, India) (pH 6.8) was prepared by dissolving di-potassium hydrogen phosphate (K_2_HPO_4_) (0.174 g) and potassium di-hydrogen phosphate (KH_2_PO_4_) (0.136 g) in 100.0 mL of distilled water and stored at room temperature. Raw milk samples were collected from the dairy farms near by Karnal and pasteurized milk samples were collected from the local market of Karnal, stored under refrigerated conditions, without further treatment before test.

### Procurement of microbial cultures

Different bacillus cultures namely *B. cereus* ATCC 13061, *B. licheniformis* ATCC 12759, *B.cereus* ATCC 11778 were procured from American Type Culture Collection (ATCC) and *B. cereus* ATCC 10876 Virginia, USA.

### General instrumentation

Inoculation and reconstitution of spore carried out in Bio-safety Level-II cabinet (Esco Biotech Pvt. Ltd., India). Incubator shaker (Eppendorf, Inc., USA.) was used for the incubation of spore. Separation of pellet done in centrifuge (Eppendorf, USA). Spore stored in − 20 °C deep freeze (Bluestar, India). Lyophilization of spores were carried out in lyophilizer (Labconco, USA). Microbiological plate reader (Perkin Elmer, USA) used for adjustment of spore O.D. Immobilization of substrate on paper strip carried out using the Micro-pipette (Eppendorf, USA).

### Preparation of spores

The production of *B. cereus* spores was carried out as per the protocol in our previous work^25^. Briefly, A single pure colony from streaked plate was transferred into 5.0 mL propagation medium and incubated at 37 °C for 24 h. 100 mL of growth medium was inoculated with overnight grown culture from propagation medium at the rate 1%, followed by incubation at 37 °C for 48 h. After incubation, culture from growth medium was further inoculated in 100 mL of sporulation medium at the rate of 7.5% for spore production. The incubation was carried out at 37 °C for 42 h followed by harvesting of spores by centrifugation at 10,000 rpm for 10 min. at 10 °C. The pellet containing spores was washed twice using potassium phosphate buffer (pH 6.8, 10 mM) by centrifugation under similar condition. The spore concentration was standardised by optimising the critical spore concentrations and volume for better colour development on the paper strip in the shortest possible time period. The produced spores were lyophilized in micro-centrifuge tubes at the optimal concentration and volume for later usage.

### Preparation and functionalization of paper strips with chromogenic substrate

Paper strips were fabricated by anchoring Whatman filter paper grade 3 on a plastic sheet with the help of two sided tape and the strips were cut in 3 cm × 0.5 cm (height x width). After cutting the strips, the working solution of nitrocfin (chromogenic substrate) was dispensed on to the paper and the strips were kept for drying at 37 °C for 30 min. Following drying, the strips were vacuum packed and stored at − 20 °C till further use [Fig. S2].

### Incorporation of germinant mixture on paper disc

Germinant solution was prepared at the optimized concentration of nutrients and solution was autoclaved (121 °C/15 min/15 lbs pressure). The germinant solution was immobilized on paper discs and discs were dried at 64 °C for 30 min. After drying, discs were collected and filled in a container and stored at 4 °C till further use [Fig. S3].

### Screening of ß-lactamase enzyme in different bacillus strains

Bacillus strains were screened for the induction of ß-Lactamase enzyme, when spores germinates in the presence of ß-lactam antibiotic residues, they will induce the production of ß-lactamase in germinating spores and this enzyme will hydrolyse the chromogenic substrate present on the strip and colour will change from yellow to red, which shoes the presence of ß-lactam antibiotic in the milk. On the other hand, when spores germinates in the absence of ß-lactam antibiotic, the induction of ß-lactamase will not occur and the chromogenic substrate will not be hydrolysed and colour will not change, which shoes the absence of ß-lactam antibiotic in the milk. Nitrocefin was used as a chromogenic substrate. As a cephalosporin, nitrocefin has a ß-lactam ring that can be hydrolysed by ß-lactamases enzyme. The degraded nitrocefin molecule quickly shifts from yellow to red after hydrolysis. Nitrocefin is a chromogenic cephalosporin, yet despite this, it doesn't seem to have any antibacterial characteristics^26^.

**Figure 1:**
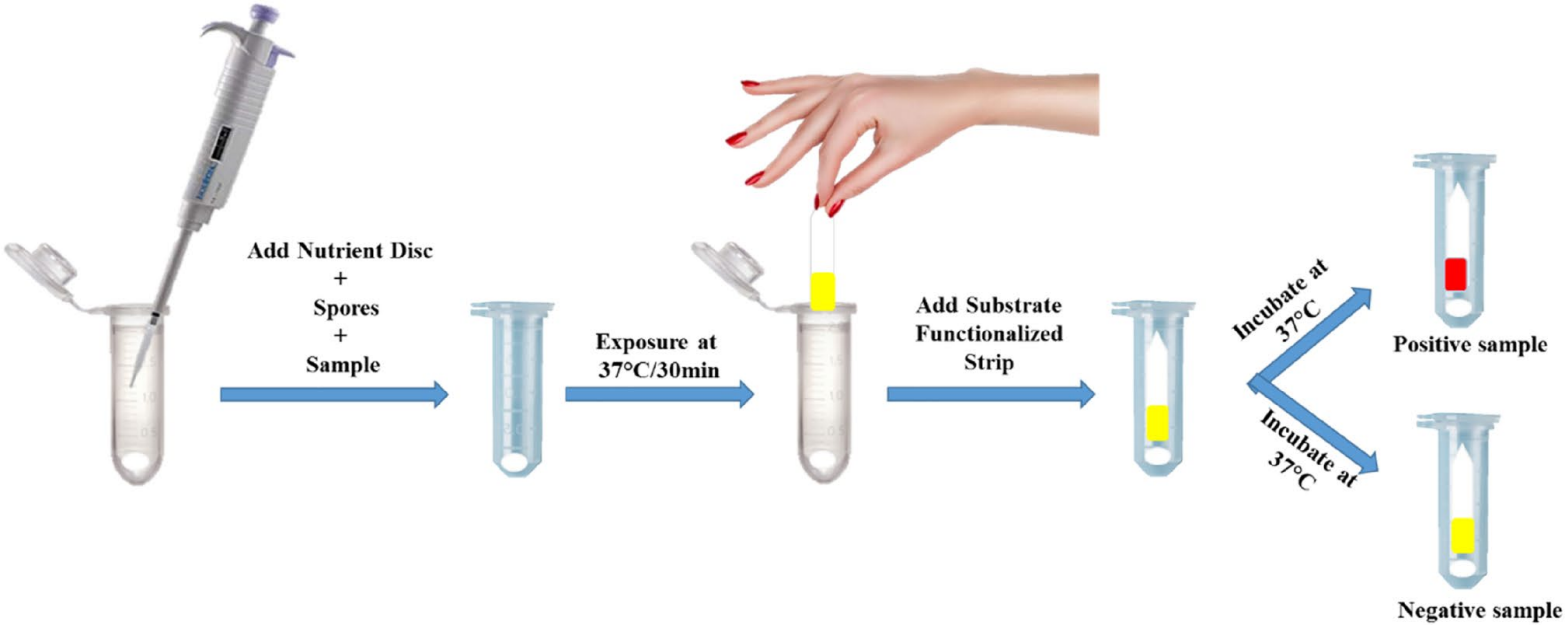
The assay procedure for detecting the ß-lactam group in milk is shown in this image. In a micro-centrifuge tube, add the nutrient disc, spores, and milk sample; vortex; and incubate at 37 °C for 30 min for the exposure step. Add the substrate-functionalized paper strip to the tube after that, and incubate once more at 37 °C for 30 min to allow the enzyme substrate reaction. Watch the strip's colour change after the incubation is over. Yellow colour indicates the absence of ß-lactam antibiotics, while red indicates the presence of ß-lactam antibiotics.

Milk sample was spiked with amoxicillin antibiotic at 10 ppb concentration to use as positive control and antibiotic free milk was used as negative control. Different bacillus strains were screened for the induction of ß-lactamase enzyme in the presence of ß-lactam antibiotics by using the protocol depicted in the Fig. 1. Bacillus strain showing yellow colour in negative control and red colour in positive control was selected for the development of the spore based paper strip biosensor for the rapid detection of ß-lactam group in milk.

### Development of spore based paper strip biosensor

The spores of *B. cereus* in the form of suspension (10–50 μL), quantity of ß-lactam antibiotic spiked milk sample (50–100 μL), quantity of chromogenic substrate (5–20 μL), exposure and incubation time (10–30 min.) were optimized for the development of spore based paper strip biosensor. The change in colour of the strip from yellow to red was taken as criteria for enzyme induction in presence of ß-lactam antibiotic residues in milk. The spore based paper strip biosensor was developed by optimizing all the essential operating parameters and assembled into a commercial kit prototype for its application in industries and under field conditions. The developed biosensor was screened for its limit of detection (LOD) against 12 different ß-lactam antibiotics such as amoxicillin, penicillin, ampicillin, carbenicillin, cloxacillin, nafcillin, oxacillin, cephalothin, cefalexin, cefoxitin, cefazolin and cefuroxime for evaluating the performance of sensor strain *B. cereus* by incubating with spiked milk samples at or below FSSAI/EU/Codex MRL^27^. To validate the results, all of the prepared antibiotic standards were evaluated with a newly developed spore based paper strip biosensor and the CHARM-ROSA test^19^. The results of validation of the sensor with 1 ppb, 2 ppb, 3 ppb, 4 ppb, and 5 ppb antibiotic standards (amoxicillin) spiked milk samples is depicted in the form of Fig. 7.

### Evaluation of spore based paper strip biosensor

Working efficacy of spore based paper strip biosensor was evaluated by interference study of non ß-lactam antibiotics (kanamycin, trimethoprim, cloramphenicol, colistin, rifampin, amikacin, tetracycline, ciprofloxacin, vancomycin, gentamycin and clindamycin) and other contaminants (Sodium hypochloride (NaClO), Disinfectant, H_2_O_2_, formalin, Aflatoxin M1, and Pesticide) in working of the developed biosensor has been done. In which different non- ß-lactam antibiotics and other contaminants has been screened at their MRL level with and without combination of ß-lactam antibiotics. Raw milk (200 nos) collected from Karnal, Haryana were also tested with the developed biosensor. Amoxicillin spiked milk at > MRL in accordance with council regulation 37/2010/EU (EC, 2010) was used as positive control while antibiotic free milk served as negative control. The result of spore based paper strip biosensor were compared with CHARM-ROSA test (Charm Science Inc. USA) and spore based assay^19,21^.

## Result and discussion

### Selection of bacillus strain as a biosensor

**Figure 2:**
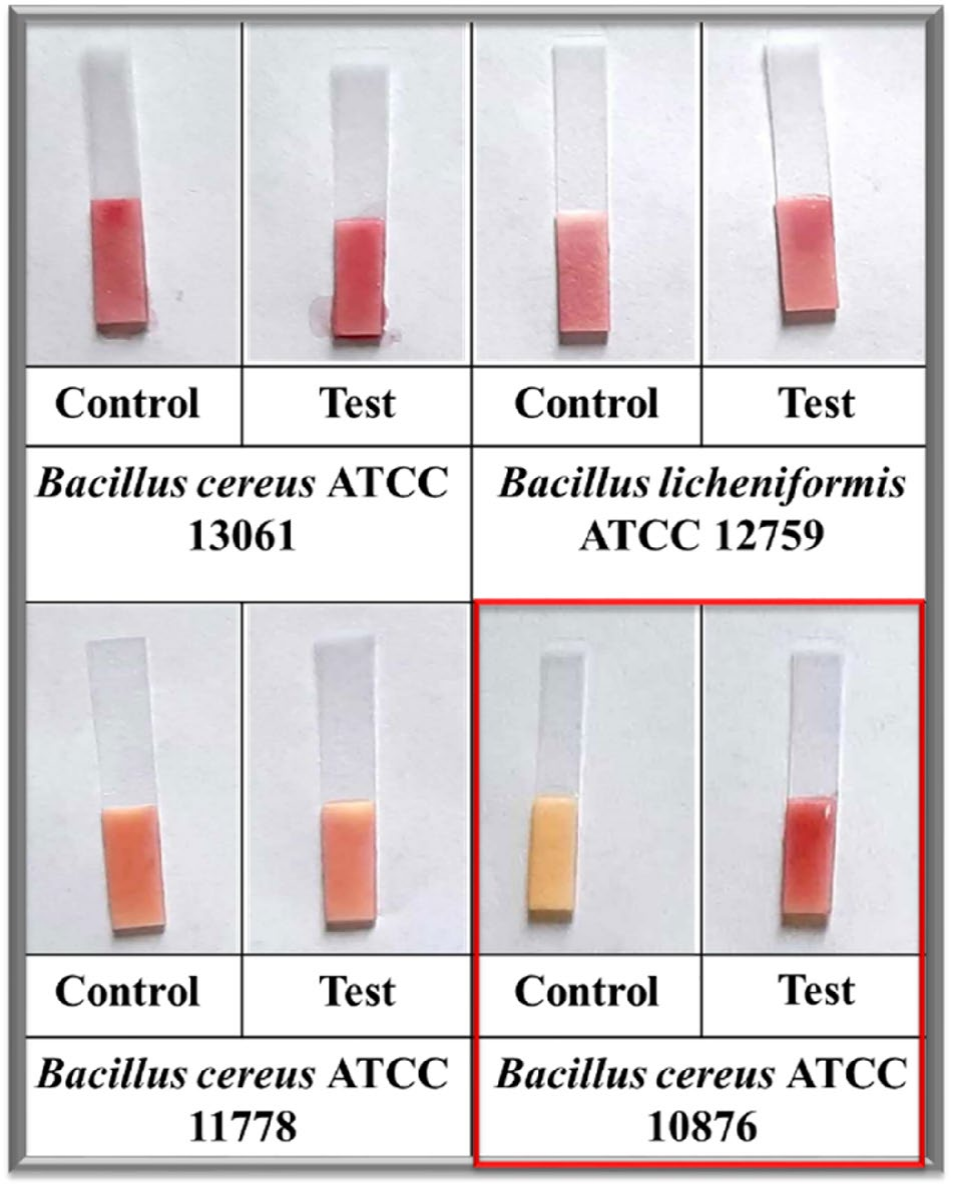
In this picture, four different bacillus cultures were screened for the induction of ß-lactamase enzyme in the presence (test) and absence (control) of ß-lactam antibiotic. Only one strain, i.e. *B. cereus* ATCC 10876 showed induction of ß-lactamase enzyme, while the other three strains produced ß-lactamase enzyme constitutively even in the absence (control) of ß-lactam antibiotic.

Four different strains of bacillus species including *B. cereus* ATCC 13061, *B. licheniformis* ATCC 12759, *B. cereus* ATCC 11778 and *B. cereus* ATCC 10876 were screened for the induction of ß-lactamase enzyme in the presence of ß-lactam antibiotics. Only one strain, *B. cereus* 10876 showed induction of ß-lactamase enzyme (colour of strip changed only in the presence of ß-lactam antibiotic), while the other three strains produced ß-lactamase enzyme constitutively even in the absence of ß-lactam antibiotic (colour of strip changed in the presence as well as in absence of ß-lactam antibiotic) as shown in Fig. 2. The *B. cereus* 10876 was chosen as the biological recognition molecule for the development of spore based paper strip biosensor for the rapid detection of the ß-lactam group in milk.

Based on the results obtained during screening of different bacillus spores (Fig. 2), it can be concluded that the expression of the ß-lactamase in *B. cereus* ATCC 10876 is inducible because the colour of the strip was changed only in the presence of ß-lactam antibiotic (test) while in the absence of ß-lactam antibiotic (control) the colour of the strip was remained yellow and the induction of ß-lactamase was also observed by Davies and his coworkers^28^. While in other strains of bacillus screened, the expression of ß-lactamase is constitutive, because the colour of the strip was changed in the presence as well as in the absence of ß-lactam antibiotic. The activity of ß-lactamase in unheated spores of *B. cereus* ATCC 10876 was achieved in real-time ranging from 50 to 60 min. However, activity of ß-lactamase in heated spores of *B. cereus* ATCC 10876 was not observed within 60 min of incubation time. Hence, based on early activity of marker enzyme, unheated spores of *B. cereus* ATCC 10876 was selected for further study and development of spore based paper strip biosensor for the specific detection of ß-lactam group in milk. Photographs of the results were taken with a smartphone in bright light, which may have contributed to colour variations on paper. Smartphones can include a variety of optical components, which can affect image quality and cause colour intensity to become more inconsistent^29^.

### Optimization of assay

In current investigation, optimization was carried out to get the best combination of all the key operating parameters for the development of the biosensor, including spore volume (10–50 µL), substrate volume (5–20 µL), sample volume containing antibiotic (50–100 µL), incubation temperature (30–45 °C), exposure time (0–30 min), and incubation time (10–30 min). All the key operating parameters were optimized by remaining others constant. The developed optimised spore based paper strip biosensor protocol for detecting ß-lactam antibiotics in milk involves two steps: exposure of bacillus spore to ß-lactam antibiotics for the induction of ß-lactamase enzyme and enzyme–substrate reaction. Each step's protocol is explained further down.

#### Step 1-exposure

20 µL spores of *B. cereus* ATCC 10876 (O.D. = 1.0 ± 0.02) were lyophilized in micro-centrifuge tubes using Labconco free zone lyophilizer. Take two tubes containing lyophilized spores and add one nutrient disc in each tube, 80 µl of negative control and positive control was added into the tubes separately. Vortex for 15 s to mix the contents of each tube. After mixing, the tubes were incubated at 37 °C for 30 min to induce expression of marker enzyme in germination spores.

#### Step 2-Enzyme substrate reaction

After exposure, the plastic strips functionalized with 10 µL of chromogenic substrate were added into each tube, which was then incubated for 30 min at 37 °C. The strips in each tube were inspected for red colour development after incubation for qualitative examination using the naked eye.

#### Result interpretation

The presence of ß-lactam antibiotics in milk was shown by the formation of red colour on the strip, whilst the absence of ß-lactam antibiotics was indicated by the yellow colour on the strip when examined with the naked eye.

### Limit of detection

**Figure 3:**
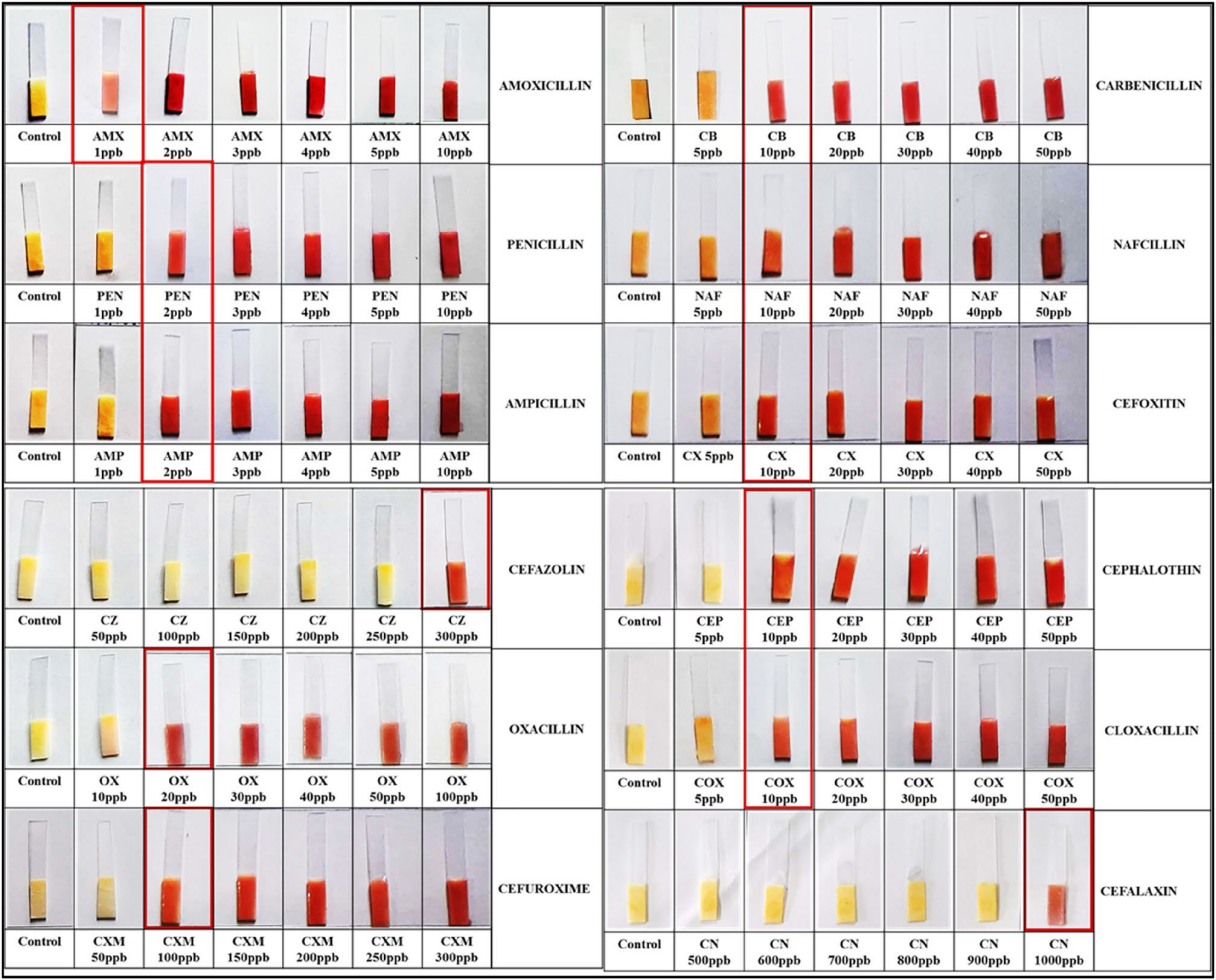
Determination of LODs of different ß-lactam antibiotics after spiking in milk at concentrations below and above their MRL including amoxicillin, penicillin, ampicillin, carbenicillin, cloxacillin, nafcillin, oxacillin, cephalothin, cefalexin, cefoxitin, cefazolin, and cefuroxime with detection limit of 1 ppb, 2 ppb, 2 ppb, 10 ppb, 10 ppb, 10 ppb, 20 ppb, 10 ppb 1000 ppb, 10 ppb 300 ppb and 100 ppb, respectively.

Limit of detection (LOD) is the lowest concentration of ß-lactam antibiotics that can induce the expression of the targeted enzyme in an enzyme induction based test. The optimized spore based paper strip biosensor was examined for LODs of 12 different ß-lactam antibiotics, The limit of detection (LOD) for various ß-lactam antibiotics, including amoxicillin, penicillin, ampicillin, carbenicillin, cloxacillin, nafcillin, oxacillin, cephalothin, cefalexin, cefoxitin, cefazolin, and cefuroxime, was determined after spiking these antibiotics in milk with detection sensitivity of 1 ppb, 2 ppb, 2 ppb, 10 ppb, 10 ppb, 10 ppb, 20 ppb, 10 ppb 1000 ppb, 10 ppb 300 ppb and 100 ppb, respectively. The minimum concentration of ß-lactam antibiotics that induced ß-lactamase enzyme induction was used to determine the LOD of that antibiotic. Milk samples were spiked with each antibiotic at concentrations below and above their MRL, then incubated at 37 °C/30 min with spores and nutrients to induce ß-lactamase production in germinating spores, followed by the addition of a chromogenic substrate functionalized strip and incubation at 37 °C/30 min as shown in Fig. 3. Antibiotic detection was also confirmed using AOAC-approved CHARM ROSA strips and a great agreement was found between these two methods.

Figure 1, describes the test methodology that was applied to determine LODs. The LOD of each ß-lactam antibiotic in the developed test was defined as the minimum concentration of ß-lactam antibiotic that generates red colour on the strip. The results of LODs of various ß-lactam antibiotics are listed in Table 1.

**Table 1:** Regulatory standards for different antibiotics and their limit of detection by spore based paper strip biosensor.

Antibiotic	MRL (ppb)	LOD (ppb)
Amoxicillin	4.0 (FSSAI)	1.0
Ampicillin	10.0 (FSSAI)	2.0
Penicillin	4.0 (FSSAI)	2.0
Carbenicillin	30.0 (EU)	10.0
Cloxacillin	30.0 (EU)	10.0
Nafcillin	30.0 (EU)	10.0
Oxacillin	30.0 (EU)	20.0
Cefalexin	10.0 (FSSAI)/100.0 (EU)	1000.0
Cephalothin	30.0 (CODEX)	10.0
Cefoxitin	–	10.0
Cefuroxime	50.0 (CODEX/EU)	100.0
Cefazoline	50.0 (EU)	300.0

### Selectivity

**Figure 4:**
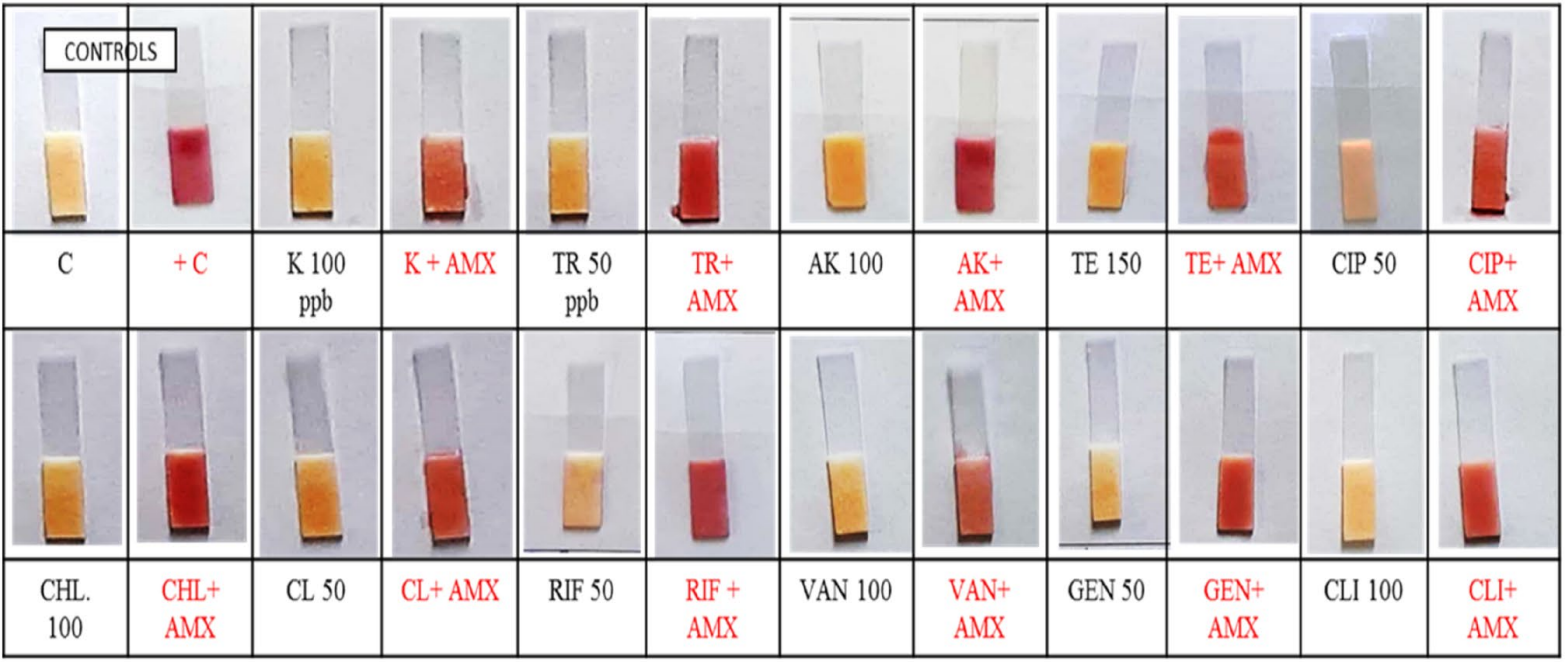
Cross-reactivity study of non-ß-lactam antibiotics on working of developed biosensor. *C* control, + *C* positive control (amoxicillin 10 ppb), *AMX* amoxicillin, *K* kanamycin, *TR* trimethoprim, *CHL* chloramphenicol, *CL* colistin, *RIF* rifampin, *AK* amikacin, *TE* tetracycline, *CIP* ciprofloxacin, *VAN* vancomycin, *GEN* gentamycin, *CLI* clindamycin.

**Figure 5:**
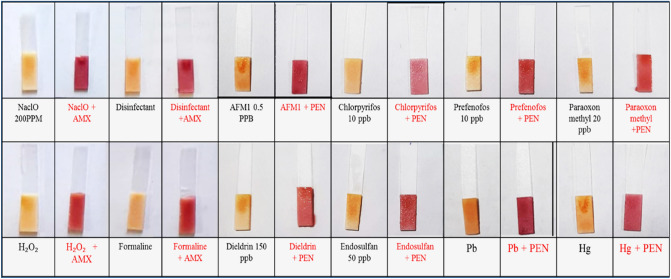
Milk contaminants like sodium hypochlorite (NaClO), disinfectant, H_2_O_2_, formalin, aflatoxin M1, pesticides (chlorpyrifos, prefenofos, paraoxon methyl, dieldrin and endosulfan) and heavy metals (Pb and Hg) were also screened at their MRL level with and without combination of β-lactam antibiotic (Amoxicillin and Penicillin).

The selectivity of developed biosensor was confirmed by cross reactivity with other toxic and non-toxic contaminants like non ß-lactam antibiotics, pesticides, aflatoxin M1, detergents and disinfectants and other contaminants most commonly found in dairy food chain. These contaminants can potentially inhibit the spore germination or enzyme activity and can hinder the overall performance of the developed biosensor. Keeping this in view, the working performance of the developed biosensor was evaluated in presence of various contaminants using optimized protocol. The significant findings are shown in Figs. 4 and 5 are described below.

Amoxicillin, kanamycin, trimethoprim, chloramphenicol, colistin, rifampin, amikacin, tetracycline, ciprofloxacin, vancomycin, gentamycin, and clindamycin were evaluated for cross-reactivity at their MRL. The results (Fig. 4) showed that none of the antibiotics mentioned above interfered with the functioning of the developed sensor. The results showed that spores are insensitive to non-ß-lactam antibiotics and the results are consistent with those of a comparable study conducted by Kumar^30^, who reported no effect of the other antibiotics on the sensors performance in detecting ß-lactam antibiotics in milk. *B. cereus* is resistant to many non- ß-lactam antibiotic^31^. The test was found working at FSSAI/CODEX/EU regulatory limits as well as lower regulatory limits with no false positive or negative results.

Pesticides takes entry into milk and other dairy products through the use of pesticides in animal feed, water, and the treatment of ectoparasites. Aside from the fact that they are harmful to human health. Pesticides can act as an inhibitor for enzyme activity and spore growth^24^. Cross-reactivity of pesticides such as chlorpyrifos, prefenofos, paraoxon methyl, dieldrin and endosulfan was tested at their MRL values in terms of inhibition of enzyme activity. The results (Fig. 5) revealed that none of the pesticides mentioned above interfered with the performance of developed biosensor. According to the findings, pesticides had no effect on spores or enzymes. The survival of *B. cereus* in the presence of pesticides has been reported by various authors^32–35^.

Aflatoxins are a family of structurally related poisonous chemicals generated by *Aspergillus flavus* and *Aspergillus parasiticus*. Humans can develop liver cancer, chronic hepatitis, jaundice, hepatomegaly, and cirrhosis as a result of aflatoxins. Aflatoxin M1 levels in milk should not exceed 50 ng/L, according to the European Union (EU) and the Codex Alimentarius Commission (CAC)^36^. Given the ubiquity of aflatoxin M1 in milk, the spore based paper strip biosensors performance was assessed in the presence of aflatoxin M1 in milk, both with and without ß-lactam antibiotics. Aflatoxin M1 had no effect on the germination and induction of *B. cereus* spores. Abdel-Shafi et al.^37^, confirmed aflatoxin M1 degradation by *B.cereus*^37^*.* Several research have been conducted to determine the presence of aflatoxin M1 in milk, and it has been determined that aflatoxin M1 is widely present in milk^36,38–41^. As a result, the spore based paper strip biosensor for detecting ß-lactam antibiotics in milk can be used in such circumstances without causing aflatoxin-M1 to interfere.

Other pollutants prevalent in milk, such as sodium hypochlorite, quaternary ammonium compound, hydrogen peroxide, and heavy metals, were evaluated with and without ß-lactam antibiotics to see if they had an inhibiting effect on the biosensor's performance. When sodium hypochlorite, quaternary ammonium compound, hydrogen peroxide, and heavy metals were used at their regulatory limits, there was no inhibitory effect on induction and enzymatic activity, as shown in Fig. 5, and the results are consistent with those of Kumar et al.^30^, who found no effect of the aforementioned contaminants on the biosensor's performance.

As a result of the findings on cross reactivity of other inhibitors on marker enzyme activity, such as non-ß-lactam antibiotics, pesticides, aflatoxin M1, sodium hypochlorite, quaternary ammonium compound, hydrogen peroxide, and heavy metals, further, it can be concluded that the induction and activity of marker enzyme is not inhibited in the presence of the aforementioned contaminants and is specific for the detection of ß-lactam antibiotic in milk. The findings are congruent with those of Kumar et al.^30^, who found that the above-mentioned pollutants had no impact on the biosensor's performance in detecting ß-lactam antibiotics in milk.

### Evaluation of spore-based paper strip biosensor

**Figure 6:**
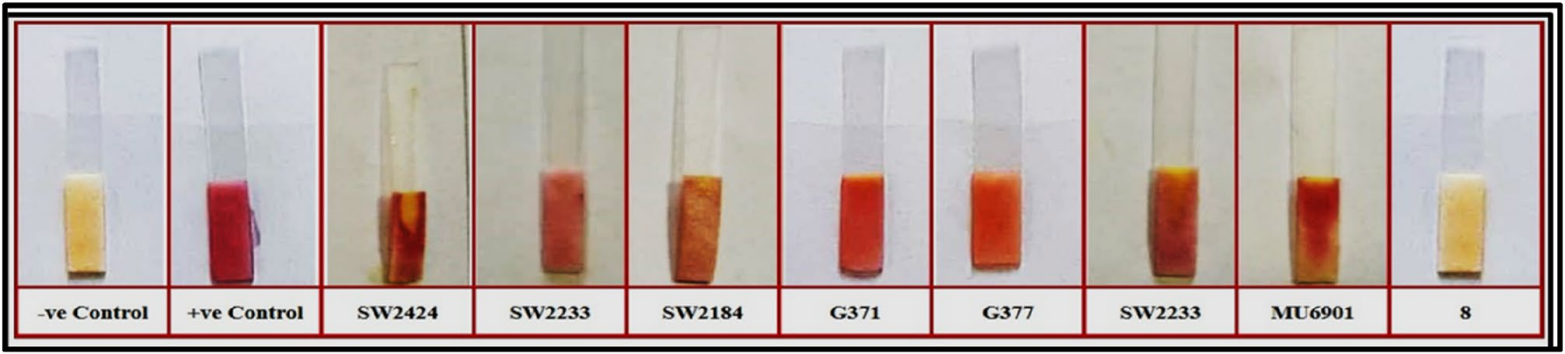
Results of evaluation of raw milk samples with spore based paper strip biosensor.

A total of 200 raw milk samples were collected from the un-organized cattle yards and evaluated for the presence of ß-lactam antibiotics using the standardized Spore-based Paper strip biosensor. The findings as showed in the Fig. 6, revealed that 7 out of 200 raw milk samples tested positive for ß-lactam antibiotics. In comparison to the data provided in the literature, the current study found a lower use of ß-lactam antibiotics in dairy production^27,42^. In this study, 3.5% of samples were found to be contaminated with ß-lactam antibiotics, whereas data from Gaare et al.^27^ and Lejaniya et al.^42^ showed a 12% ß-lactam antibiotic contamination in raw milk.

A total of 105 pasteurized milk samples from various brands were purchased from local market of Karnal and tested for the presence of ß-lactam antibiotics using the standardized spore-based paper strip biosensor. The result findings revealed that none of the pasteurized milk samples tested positive for ß-lactam antibiotics (Table 2). In compared to published statistics, the current investigation discovered that ß-lactam antibiotics are used less frequently in organized milk production. ß-lactam antibiotic contamination was discovered in 0% of pasteurized milk samples in this investigation, however data from Gaare et al.^27^ showed a 4% ß-lactam antibiotic contamination in pasteurized milk.

**Table 2:** Evaluation of spore based paper strip biosensor under field conditions.

No of samples tested	Positive for ß-lactam (Spore based paper strip biosensor)	Positive for ß-lactam (CHARM-ROSA test)
Raw milk 200	7	7
Pasteurized milk 105	0	0

### Validation of spore-based paper strip biosensor

**Figure 7:**
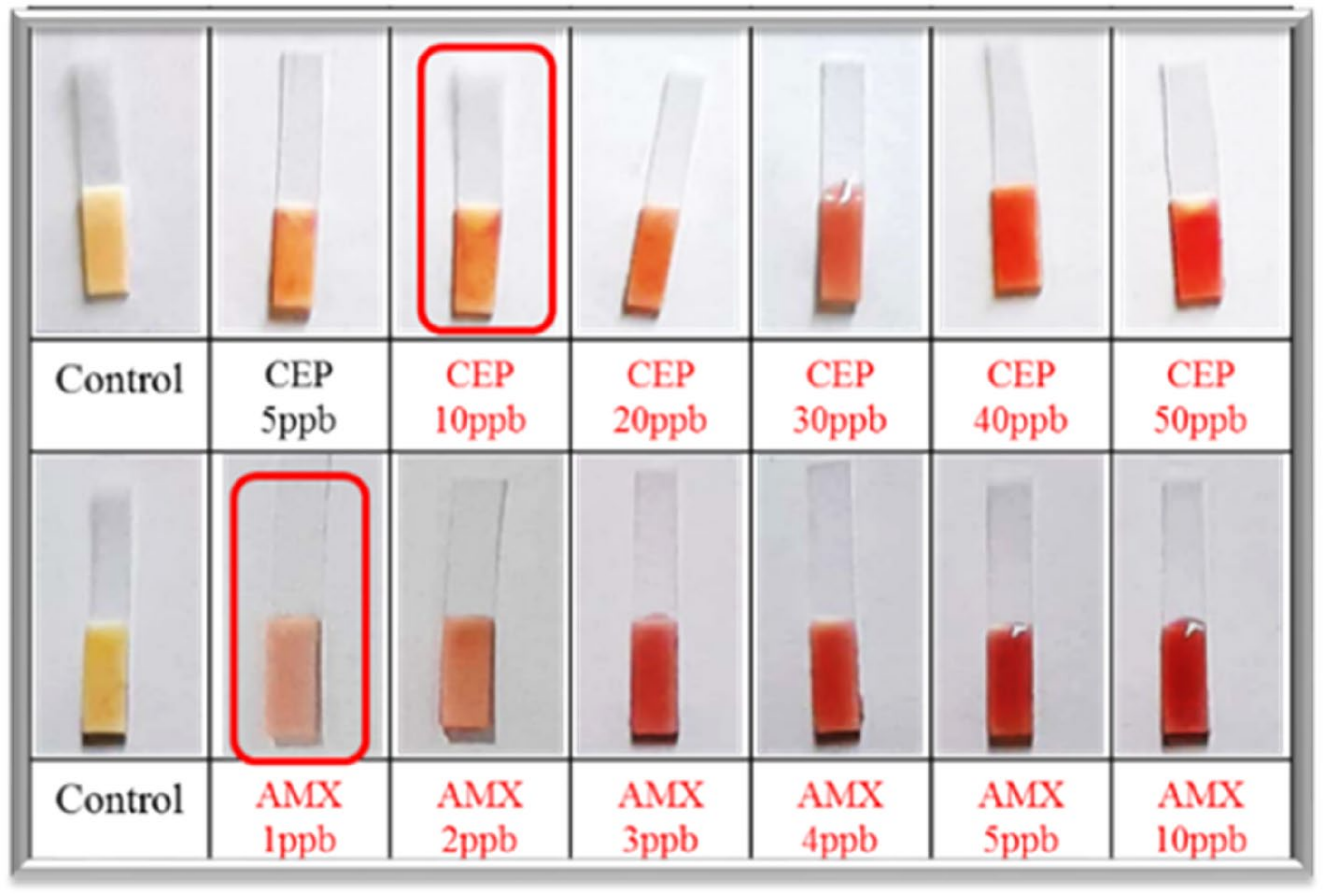
Validation of spore based paper strip biosensor with CHARM-ROSA test using spiked milk samples.

Reliable analytical data are needed for proper assessment of hazardous findings in the evaluation of scientific investigations as well as in everyday practice. Unreliable analytical data may result in unjustified legal repercussions for the defendant or incorrect commodity treatment. As a result, new analytical methods for use in the laboratory or in the field typically require method development and extensive validation. This is particularly relevant in the case of quality management and standardization, which have recently become more important in analytical toxicology^43^. In this part, validation of spore-based paper strip biosensor will be presented. The ß-lactam antibiotics spiked milk standards and developed spore-based paper strip biosensor was validated with CHARM-ROSA test for detection of ß-lactam antibiotics in milk (Table 3, Fig. 7).

**Table 3:** Validation of the standards with CHARM-ROSA test.

Amoxicillin			Cephalothin		
Concentration (ppb)	Results	Reading	Concentration (ppb)	Results	Reading
0	− ve	− 0662	0	− ve	− 0655
1	+ ve	+ 0758	1	− ve	− 1249
2	+ ve	+ 1877	2	+ ve	+ 1468
3	+ ve	+ 2365	3	+ ve	+ 1925
4	+ ve	+ 2973	4	+ ve	+ 2605
5	+ ve	+ 3635	5	+ ve	+ 2622

### Validation of the standards prepared by spiking ß-lactam in milk

Milk was spiked with ß-lactam antibiotics to prepare standards^13^, and the prepared ß-lactam antibiotics standards were tested with the CHARM-ROSA ß-lactam strip test to authenticate the study's ß-lactam antibiotics standards. Simultaneously all the standards were also tested with the developed spore-based paper strip biosensor. Results obtained in this study are listed in the Table 3 and Fig. 7. According to the findings of this investigation (Fig. 7) the developed spore-based paper strip biosensor has a sensitivity of 1 ppb for amoxicillin and 10 ppb for cephalothin.

### Validation with natural milk samples

**Figure 8:**
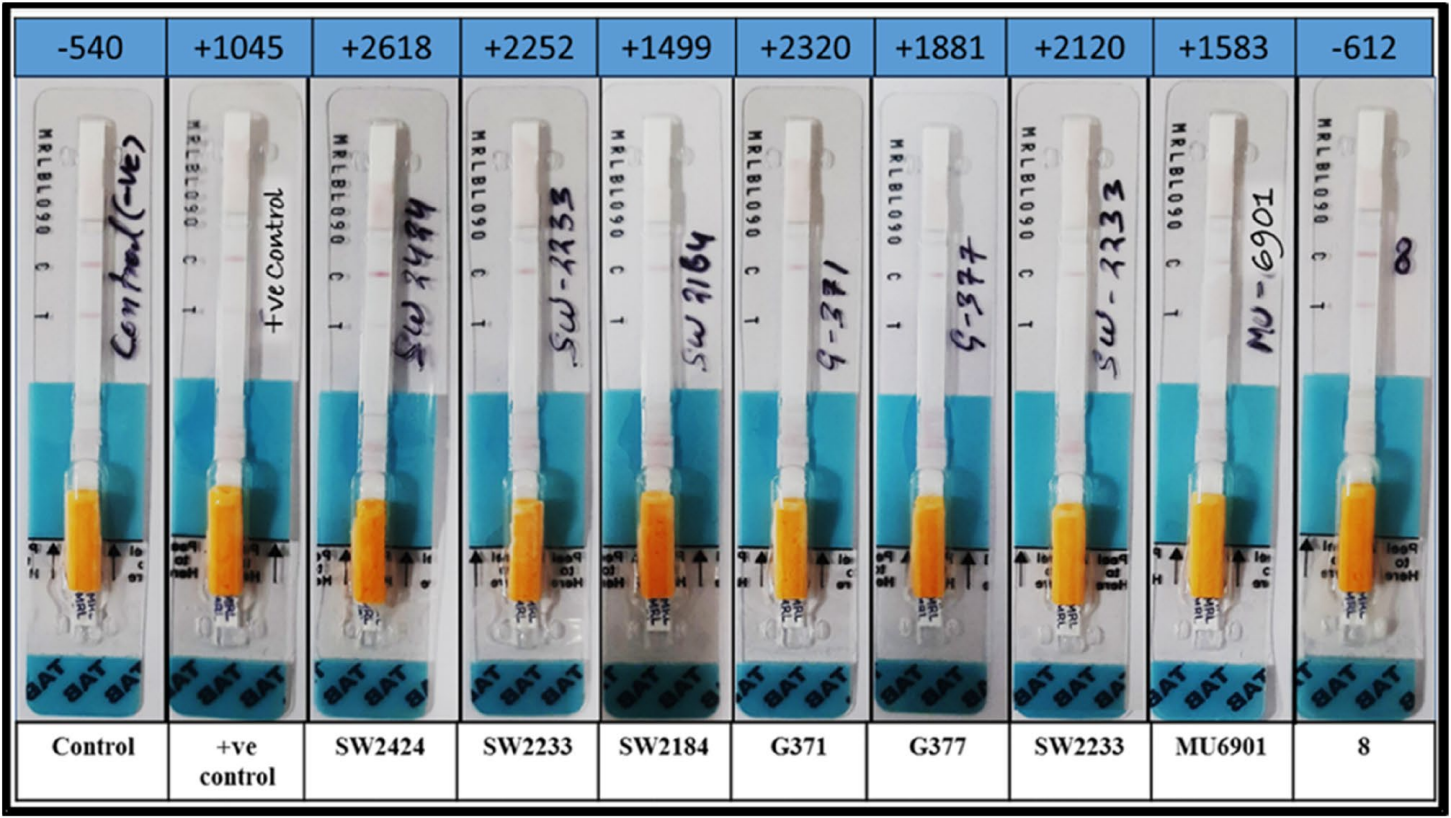
This picture showing the results of CHARM-ROSA ß-lactam strip test with their reading on top.

The CHARM-ROSA ß-lactam strip test was used to test 200 samples obtained for the evaluation study, and 7 samples that were positive with the developed spore-based paper strip biosensor were likewise positive with the CHARM-ROSA ß-lactam strip test (Fig. 8). The results of this study shows that the CHARM-ROSA ß-lactam strip test has a perfect correlation with the spore-based paper strip biosensor that was developed.

### Shelf stability

**Figure 9:**
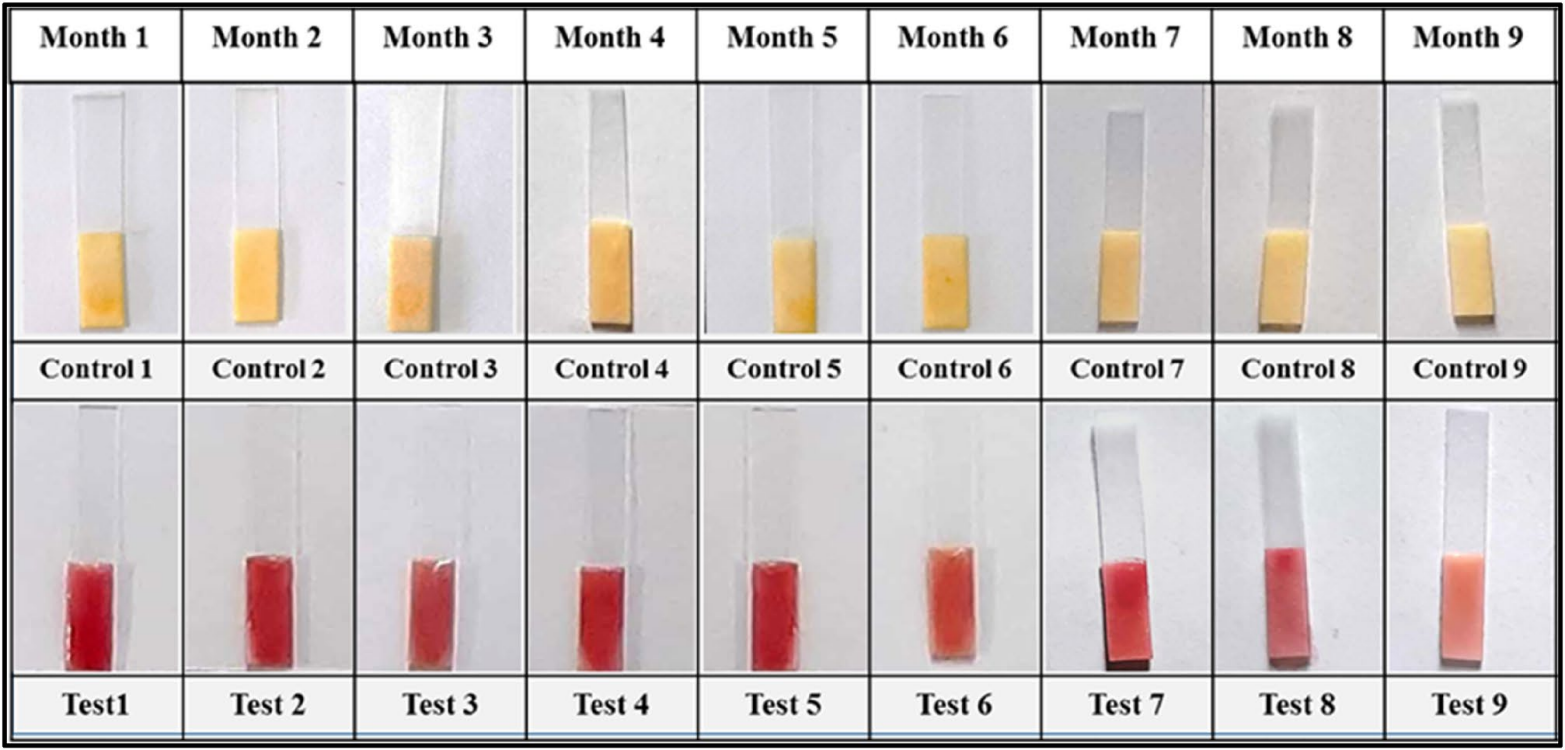
Shelf life stability of lyophilized spores at 4 °C, shows the best colour intensity up to 9 months of storage.

The shelf stability of bacillus spores is critical for identifying their capability in inducing and expressing marker enzyme activity during storage settings in order to obtain consistent and accurate results across time. 200 tubes of lyophilized spores were manufactured for this purpose and stored at 4 °C. According to the optimized protocol, the shelf stability of lyophilized spores stored at 4 °C was assessed in terms of induction and expression of ß-lactamase enzyme and its activity at 30 day intervals for a period of 6–9 months. As a result, when stored at 4 °C, the lyophilized spores were shown to be active for up to 9 months, as evidenced by the induction and production of the ß-lactamase enzyme, as well as its activity as evidenced by the formation of red colour (Fig. 9). However, prolonged storage of spores reduced the induction, expression, and activity of the ß-lactamase enzyme, but had no effect on the performance of the sensor. The shelf stability obtained in this investigation is higher than what has previously been reported in the literature^12,13^. As a result, in this work, using spore as a source of marker enzyme is a good way to make the sensor shelf-stable.

**Figure 10:**
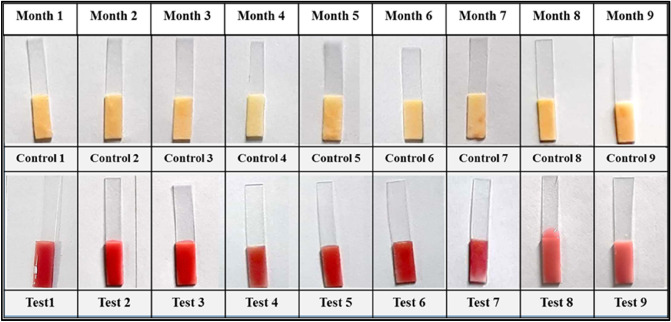
Shelf life stability of vacuum packed, substrate functionalized paper strip stored at − 20 °C, shows the best colour intensity up to 9 months of storage.

**Figure 11:**
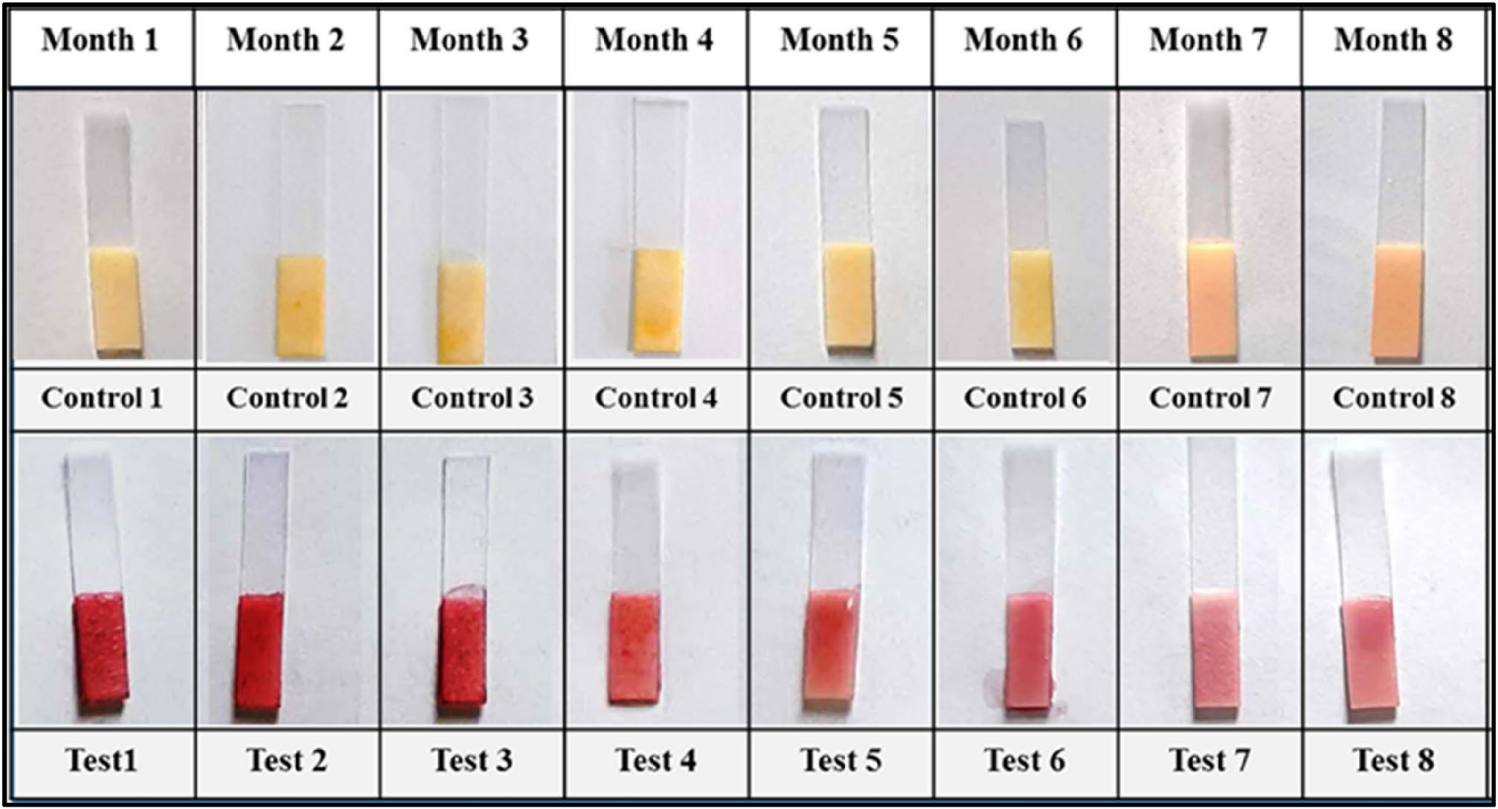
Shelf life stability of vacuum packed, substrate functionalized paper strip stored at − 4 °C, shows the best colour intensity up to 8 months of storage.

The stability of substrate functionalized paper strips holds key importance in working of the developed assay. In order to get constant and reliable results, paper strips were functionalized with chromogenic substrate and few packets of paper strips were vacuum packed and stored at − 20 °C and − 4 °C. The working of the prepared strips was checked for a period of 6–9 months (at 1 month time interval). As a result, paper strips stored at − 20 °C are stable up to 9 months (Fig. 10), whereas the strips stored at − 4 °C are stable up to 8 months (Fig. 11), as evidenced by the formation of red colour. However, long-term storage of substrate functionalized paper strips, on the other hand, resulted in a reduction in colour intensity but had no effect on the performance of developed biosensor. The shelf stability of substrate functionalized paper strips obtained in this study is better than what has been reported previously in the literature^44^. As a consequence, vacuum packaging of the substrate functionalized paper strips is an excellent technique to keep the sensor shelf-stable for a long period.

## Conclusion

A spore-based paper strip biosensor that can detect the ß-lactam group of antibiotics in milk has been developed using an enzyme induction concept. The designed biosensor can detect twelve distinct ß-lactam antibiotics, including penicillin and cephalosporins. Antibiotics were discovered in milk, confirming the technology's potential for real-time analysis. This is also the first time that spore-based enzyme induction has been demonstrated to be a reliable detection method for the ß-lactam group in milk, contributing in the fight against microbial drug resistance. Because it can detect the ß-lactam group of antibiotics, in milk within one hour, the new spore-based paper strip biosensor has the potential to revolutionise the point-of-care diagnostics sector for animal healthcare and antimicrobial resistance prevention in humans. For the ß-lactam group of antibiotics suggested by FSSAI/CODEX, the sensitivity of the developed biosensor is below MRL. The working of the developed test has been found to be free of non-ß-lactam antibiotics and other pollutants. The spore-based paper strip biosensor is inexpensive and very sensitive to antibiotics in the ß-lactam class. By visual inspection, the current technique detects the ß-lactam group of antibiotics semi-quantitatively up to LOD level, which is highly useful for field application. The FSSAI has also recommended the harmonisation of CODEX criteria for antibiotic residues in milk, which will need testing of milk at many phases of production, processing, distribution, and storage. The spore-based paper strip biosensor created has commercial promise in the dairy industry because it is low-cost, sensitive, selective, repeatable, and real-time. It is a low-cost alternative to currently used conventional methods. The created technology is truly innovative on a worldwide scale, as it is unique and superior than prior art in terms of cost per test and infrastructure required for testing.

## Supplementary Information

Supplementary Information.

## Data Availability

All data generated or analysed during this study are included in this published article [and its supplementary information file].

## References

[1] Martinez AW, Philli ST, Butte MJ, Whitesides GM (2007). Patterned paper as a platform for inexpensive, low‐volume, portable bioassays. Angewandte Chemie.

[2] Yetisen AK, Akram MS, Lowe CR (2013). Based microfluidic point-of-care diagnostic devices. Lab Chip.

[3] Clark LC, Lyons C (1962). Electrode systems for continuous monitoring in cardiovascular surgery. Ann. N. Y. Acad. Sci..

[4] Herold KE, Rasooly A, Sandwall P (2013). Biosensors and molecular technologies for cancer diagnostics. Med. Phys..

[5] Tetyana P, Shumbula PM, Njengele-Tetyana Z (2021). Biosensors: Design, Development and Applications.

[6] Suntornsuk W, Suntornsuk L (2020). Recent applications of paper-based point-of-care devices for biomarker detection. Electrophoresis.

[7] Chambers JP, Arulanandam BP, Matta LL, Weis A, Valdes JJ (2008). Biosensor recognition elements. Curr. Issues Mol. Biol..

[8] Tehri, N. *Spore Based Sensor for Pesticide Residues in Milk *(Doctoral dissertation, NDRI) (2015).

[9] Gopaul, R. *Paper Strip Based Spore Sensor for Pesticide Residues in Milk* (Doctoral dissertation, NDRI) (2015).

[10] Morab, S. *Evaluation and Validation of Paper-Strip Sensor for Detection of Pesticide Residues in Milk *(Masters Dissertation, NDRI) (2016).

[11] Kumar, N. *et al*. Rapid spores-enzyme based miniaturized assay for detection of pesticide residues. Indian Patent Application No. 3819/DEL/2015 (2015)

[12] Karanpriya. *Paper Strip Based Assay for Detection of Heavy Metals in Milk* (Doctoral dissertation, NDRI, Karnal) (2020).

[13] Anand, S. P. *Evaluation and Validation of Strip Based Sensor for Rapid Detection of Antibiotics in Milk* (Doctoral dissertation, NDRI) (2017).

[14] Allen HK, Levine UY, Looft T, Bandrick M, Casey TA (2013). Treatment, promotion, commotion: Antibiotic alternatives in food-producing animals. Trends Microbiol..

[15] Mitchell JM, Griffiths MW, McEwen SA, McNab WB, Yee AJ (1998). Antimicrobial drug residues in milk and meat: Causes, concerns, prevalence, regulations, tests, and test performance. J. Food Prot..

[16] WHO-AGISAR. *Critically Important Antimicrobials for Human Medicine—5th Revision 2016* (2017).

[17] TOI. Tolerance Limits’ to Be Fixed by Food Regulator for Presence of Antibiotics in Animal, Foods. https://www.fssai.gov.in/upload/media/FSSAINewsAntibioticsTOI01.08.2018.pdf. Accessed 12 June 2019.

[18] Liana DD, Raguse B, Gooding JJ, Chow E (2012). Recent advances in paper-based sensors. Sensors.

[19] Kumar N (2021). Understanding antibiotic usage on small-scale dairy farms in the Indian states of Assam and Haryana using a mixed-methods approach—Outcomes and challenges. Antibiotics.

[20] Kumar, N., Patil, G. R., Rane, S. & Malik, R. K. *A Novel Process of Sporulation, Activation and Germination in Thermophilic Bacteria for Rapid Detection of Antibiotic Residues in Milk*. *Indian Patent No. 264145* (2006)*.*

[21] Kumar N, Raghu HV, Kumar A, Haldar L, Khan A, Rane S, Malik RK (2012). Spore germination based assay for monitoring antibiotic residues in milk at dairy farm. World J. Microbiol. Biotechnol..

[22] Kumar, N. *et al*. *Development of Enzyme-Spore Based Assay (s) for Monitoring Antibiotic Residues in Milk*. Indian Patent No. 365074 (2014)*.*

[23] Kumar, N., Das, S. & Gaare, M. *Spore Germination Based Detection Kit for ß-Lactam Group in Milk*. Indian Patent No. 273160 (2009a).

[24] Dasriya V, Joshi R, Ranveer S, Dhundale V, Kumar N, Raghu HV (2021). Rapid detection of pesticide in milk, cereal and cereal based food and fruit juices using paper strip-based sensor. Sci. Rep..

[25] Singh NA, Kumar N, Raghu HV, Sharma PK, Singh VK, Khan A, Raghav N (2013). Spore inhibition-based enzyme substrate assay for monitoring of aflatoxin M1 in milk. Toxicol. Environ. Chem..

[26] O'Callaghan CH, Morris A, Kirby SM, Shingler AH (1972). Novel method for detection of β-lactamases by using a chromogenic cephalosporin substrate. Antimicrob. Agents Chemother..

[27] Gaare M, Kumar N, Raghu HV, Khan A, Singh VK (2012). Specific detection of β-lactam antibiotics in milk by spore based assay. Int. Res. J. Microbiol..

[28] Davies RB, Abraham EP, Melling J (1974). Separation, purification and properties of β-lactamase I and β-lactamase II from *Bacillus cereus* 569/H/9. Biochem. J..

[29] Barbosa AI, Gehlot P, Sidapra K, Edwards AD, Reis NM (2015). Portable smartphone quantitation of prostate specific antigen (PSA) in a fluoropolymer microfluidic device. Biosens. Bioelectron..

[30] Kumar, S. *Development of Chromogenic Based Assay & Kit Prototype for Specific Detection of ß-Lactam Group in milk* (Masters Dissertation, NDRI) (2009b).

[31] Fiedler G (2019). Antibiotics resistance and toxin profiles of *Bacillus cereus*-group isolates from fresh vegetables from German retail markets. BMC Microbiol..

[32] Narayanan M, Kumarasamy S, Ranganathan M, Kandasamy S, Kandasamy G, Gnanavel K (2020). Enzyme and metabolites attained in degradation of chemical pesticides ß Cypermethrin by *Bacillus cereus*. Mater. Today Proc..

[33] Zhang H (2016). Biodegradation potential of deltamethrin by the *Bacillus cereus* strain Y1 in both culture and contaminated soil. Int. Biodeterior. Biodegrad..

[34] Singh B, Kaur J, Singh K (2012). Biodegradation of malathion by *Brevibacillus sp.* strain KB2 and *Bacillus cereus* strain PU. World J. Microbiol. Biotechnol..

[35] Liu, Z. Y., Chen, X., Shi, Y. & Su, Z. C. Bacterial degradation of chlorpyrifos by *Bacillus cereus*. In *Advanced Materials Research* Vol. 356 (ed. Li, H. Xu, Q. & Zhang, D.) 676–680 (Trans Tech Publications Ltd, 2012).

[36] Darsanaki RK, Mohammadi M, Kolavani MH, Issazadeh K, Aliabadi MA (2013). Determination of aflatoxin M1 levels in raw milk samples in Gilan, Iran. Adv Stud Biol.

[37] Abdel-Shafi S, Shehata S, Shindia A, El-Meligy K, Khidr A (2018). Biodegradation of aflatoxins by bacteria, Egypt. J. Microbiol..

[38] Sumon AH (2021). The presence of Aflatoxin M1 in milk and milk products in Bangladesh. Toxins.

[39] Muaz K (2022). Aflatoxin M1 in milk and dairy products: Global occurrence and potential decontamination strategies. Toxin Rev..

[40] Thukral H, Dhaka P, Bedi JS, Aulakh RS (2022). Occurrence of aflatoxin M1 in bovine milk and associated risk factors among dairy farms of Punjab, India. World Mycotoxin J..

[41] Sharma H, Jadhav VJ, Garg SR (2020). Aflatoxin M1 in milk in Hisar city, Haryana, India and risk assessment. Food Addit. Contam. Part B.

[42] Lejaniya A, Sathya P, Sathian C, Anil KS, Geetha R, Radha K (2017). Screening of pooled milk samples for ß-lactam and tetracycline antibiotic residue. Int. J. Sci. Environ. Technol..

[43] Peters FT, Drummer OH, Musshoff F (2007). Validation of new methods. Forensic Sci. Int..

[44] Boehle KE, Carrell CS, Caraway J, Henry CS (2018). Paper-based enzyme competition assay for detecting falsified β-lactam antibiotics. ACS Sens..

