# Supplementary material for: Spore-based innovative paper-strip biosensor for the rapid detection of ß-lactam group in milk
Source: Sci Rep. 2022 Dec 19;12:21965. doi: 10.1038/s41598-022-26466-7 (PMC9763390; doi:10.1038/s41598-022-26466-7)
Supplement: Supplementary file 1 — Supplementary Information. [file 41598_2022_26466_MOESM1_ESM.pdf]

Spore-Based Innovative Paper-Strip Biosensor for the Rapid Detection of ß-Lactam Group in Milk

Prashant Goel[1], Raghu H. V[1]. and Naresh Kumar[1]*,

[1]*Dairy Microbiology Division, ICAR-National Dairy Research Institute, Karnal, India.*

** Corresponding Authors: Dr. Naresh Kumar nkg6825@gmail.com)*

(Prashant Goel, prashant318goyal@gmail.com; Dr. Naresh Kumar, nkg6825@gmail.com; Dr. Raghu H V, 4rvsy.dmndri@gmail.com)

Supplementary information:

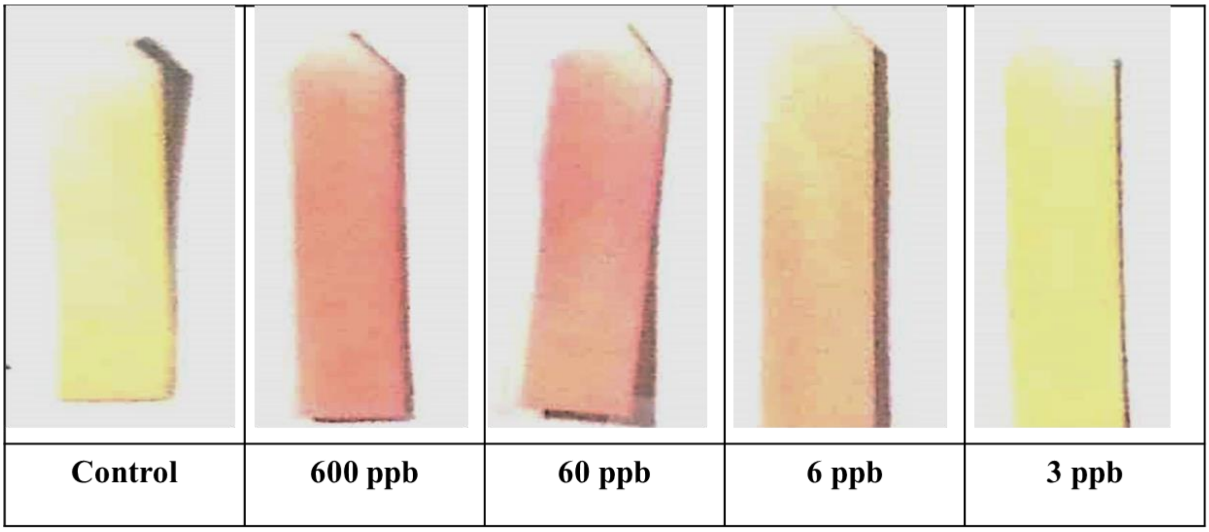

Supplementary Figure S1. Evaluation of proof of concept for detection of ß-lactam group based on induction principle

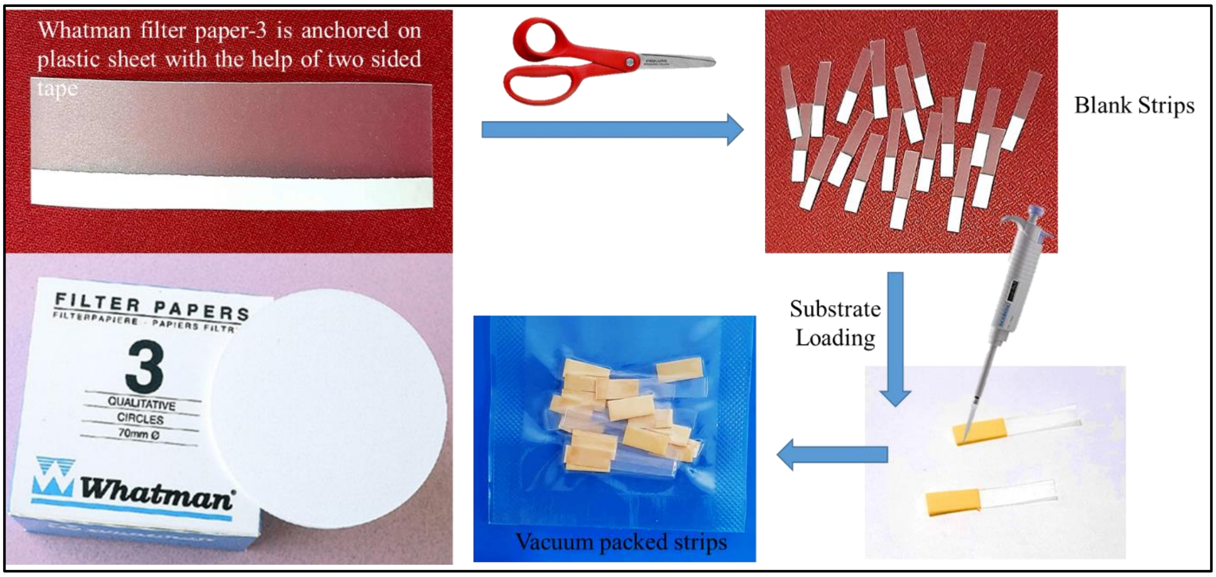

Supplementary Figure S2. Designing and fabrication of paper-strip

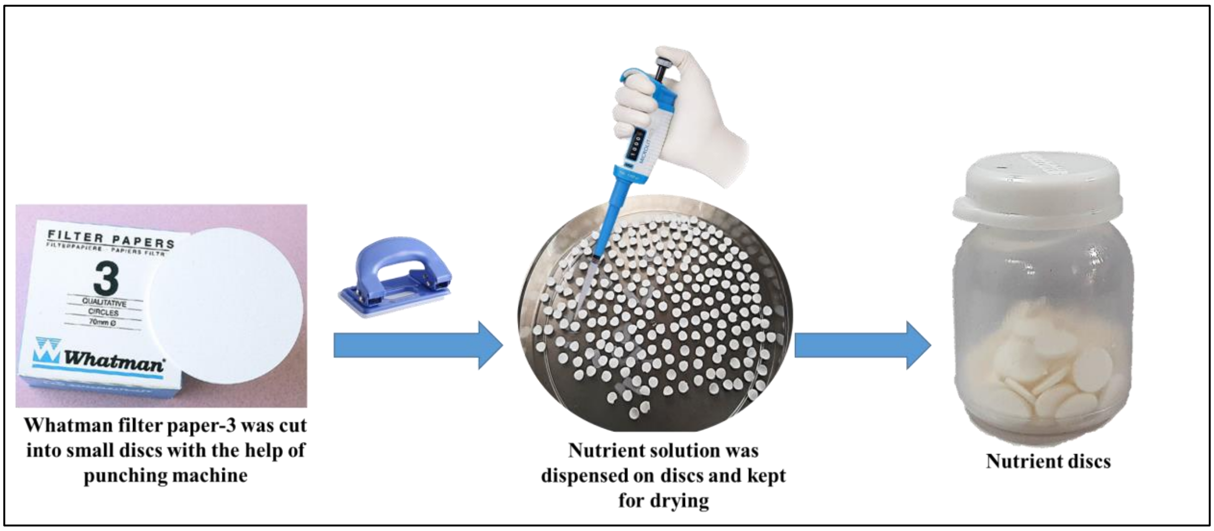

Supplementary Figure S3. Preparation of Nutrient discs

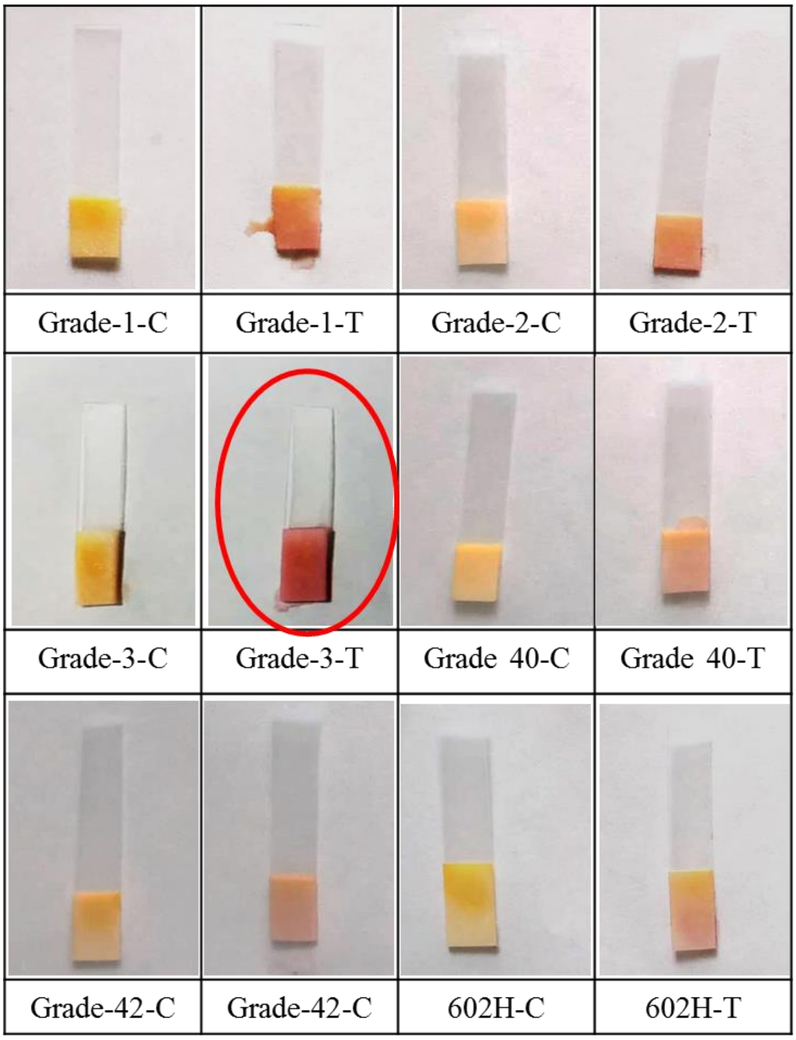

Supplementary Figure S4. Screening of paper for assay development

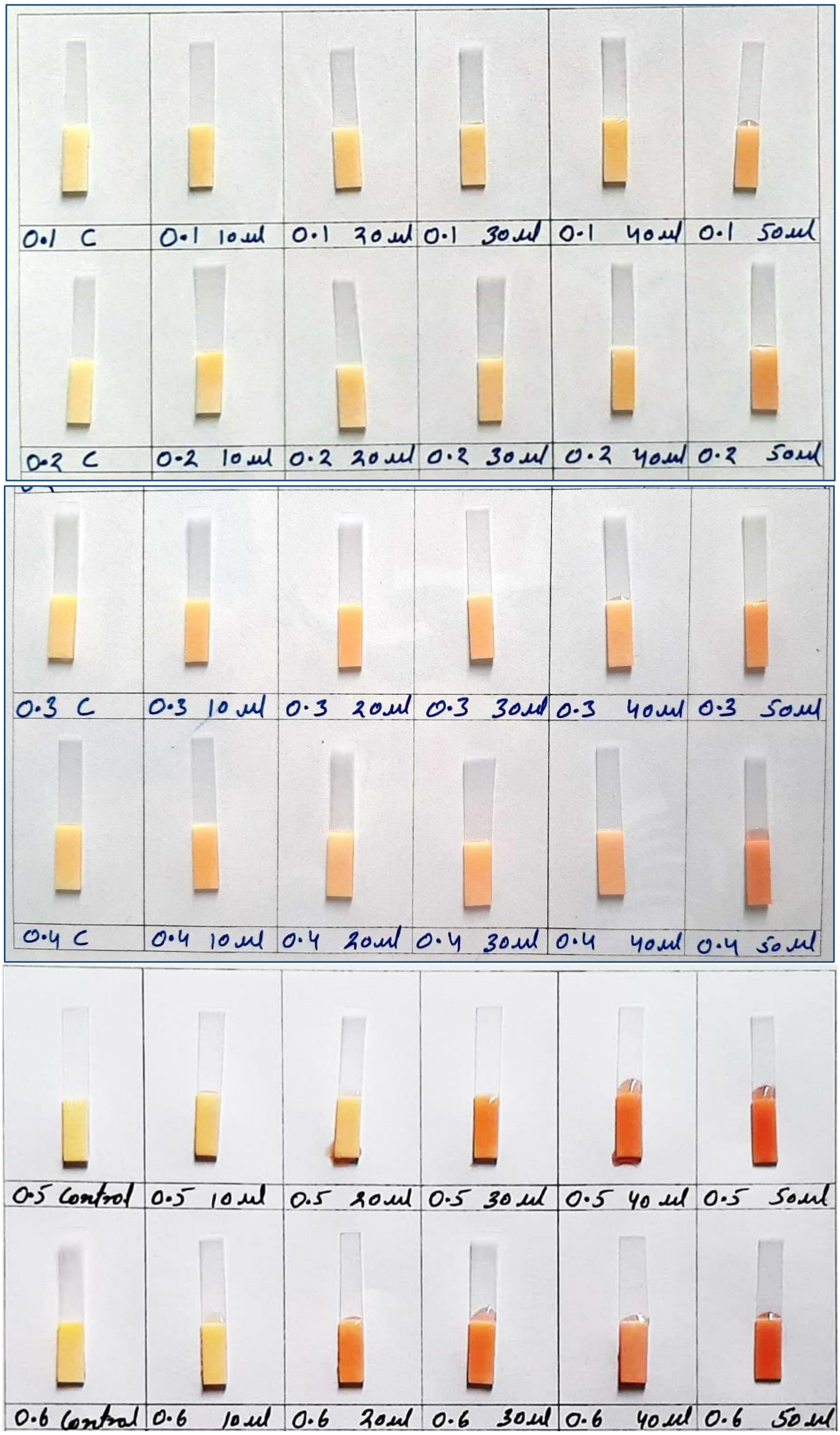
0.1 C
0.1 10 µl
0.1 20 µl
0.1 30 µl
0.1 40 µl
0.1 50 µl
0.2 C
0.2 10 µl
0.2 20 µl
0.2 30 µl
0.2 40 µl
0.2 50 µl
0.3 C
0.3 10 µl
0.3 20 µl
0.3 30 µl
0.3 40 µl
0.3 50 µl
0.4 C
0.4 10 µl
0.4 20 µl
0.4 30 µl
0.4 40 µl
0.4 50 µl
0.5 Control
0.5 10 µl
0.5 20 µl
0.5 30 µl
0.5 40 µl
0.5 50 µl
0.6 Control
0.6 10 µl
0.6 20 µl
0.6 30 µl
0.6 40 µl
0.6 50 µl

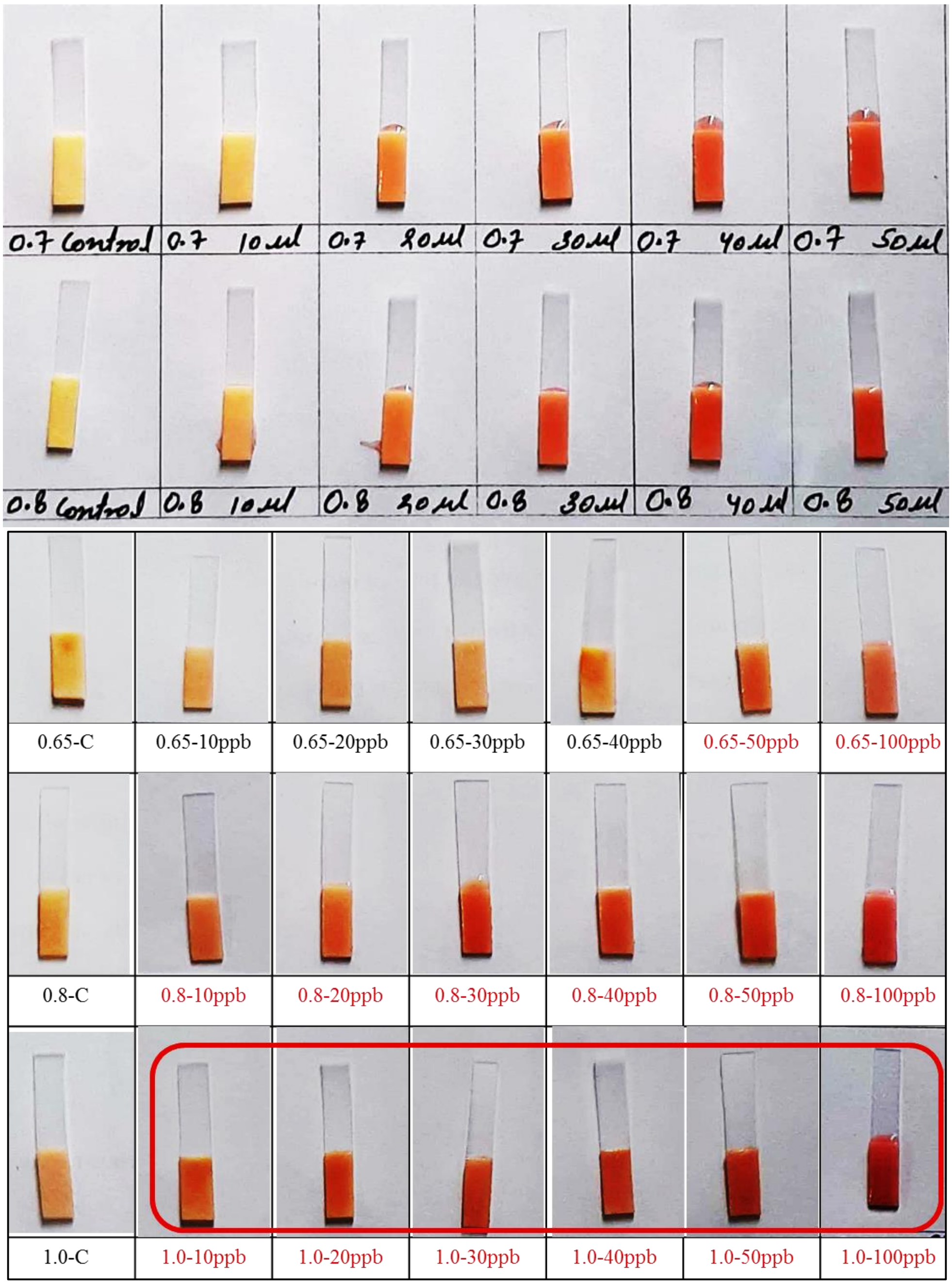

Supplementary Figure S5. Optimization of the Spore volume at different OD

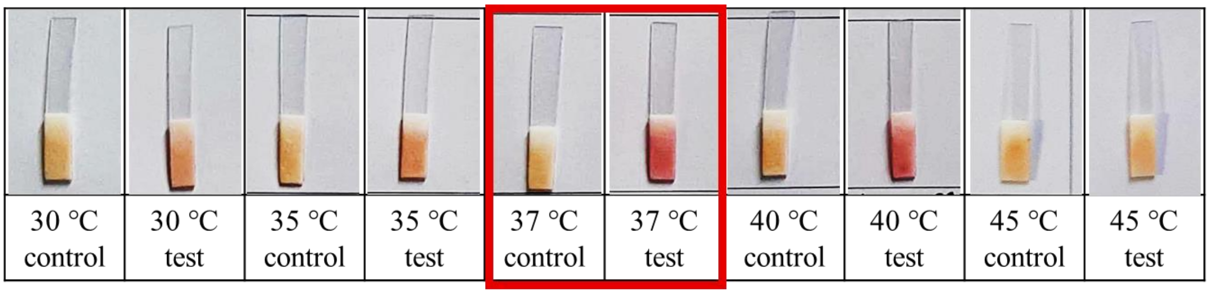

Supplementary Figure S6. Optimization of incubation temperature

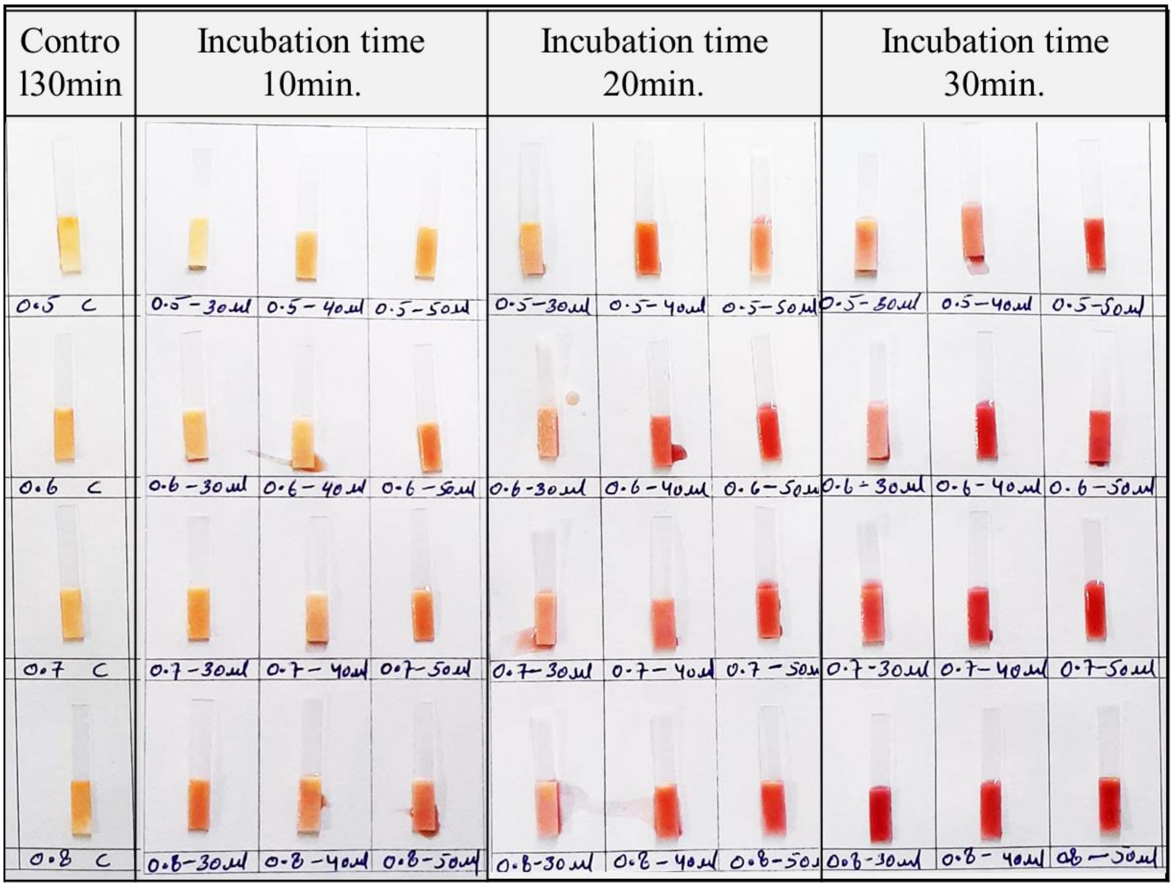

Supplementary Figure S7. Optimization of Incubation Time at Different Spore volume at different OD

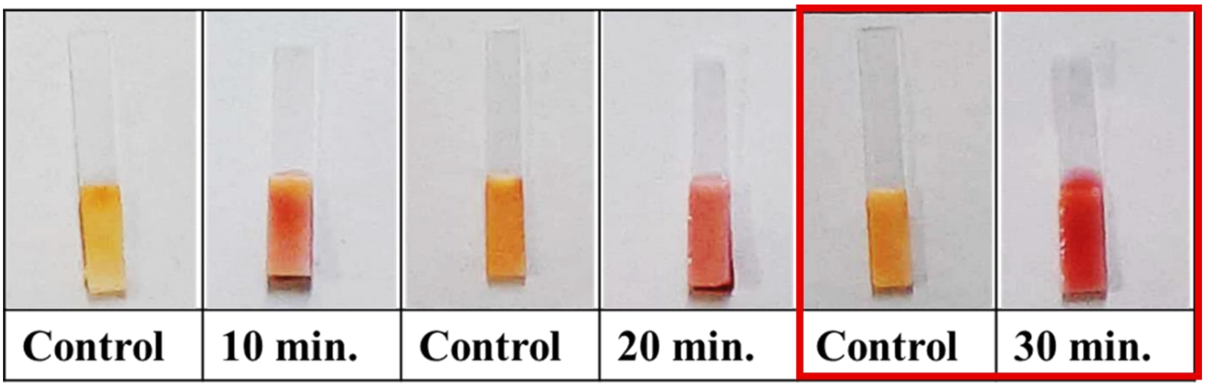

Supplementary Figure S8. Optimization of Incubation Time

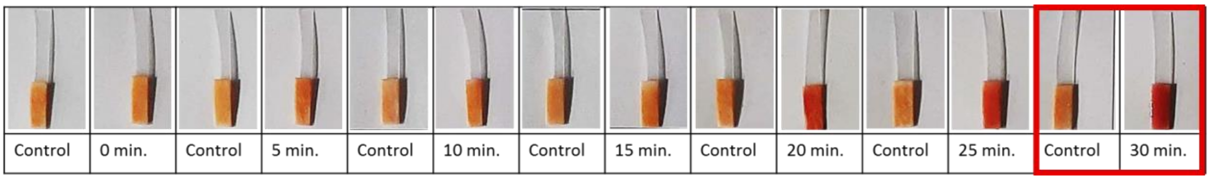

Supplementary Figure S9. Optimization of Exposure Time

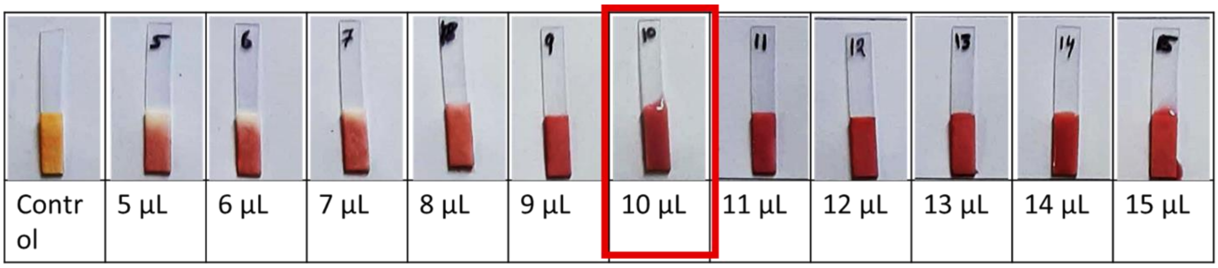

Supplementary Figure S10. Optimization of Substrate Volume

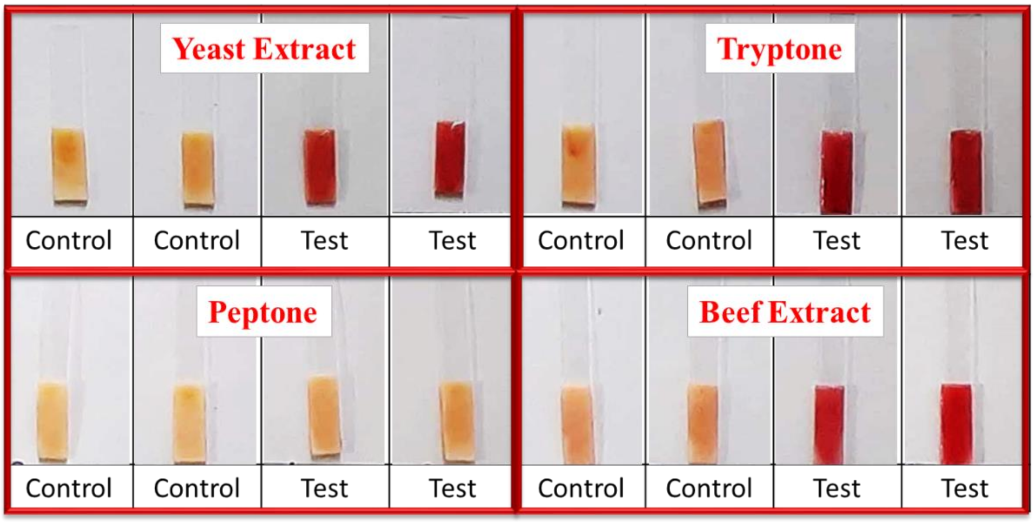

Supplementary Figure S11. Screening of Nutrients for induction assay

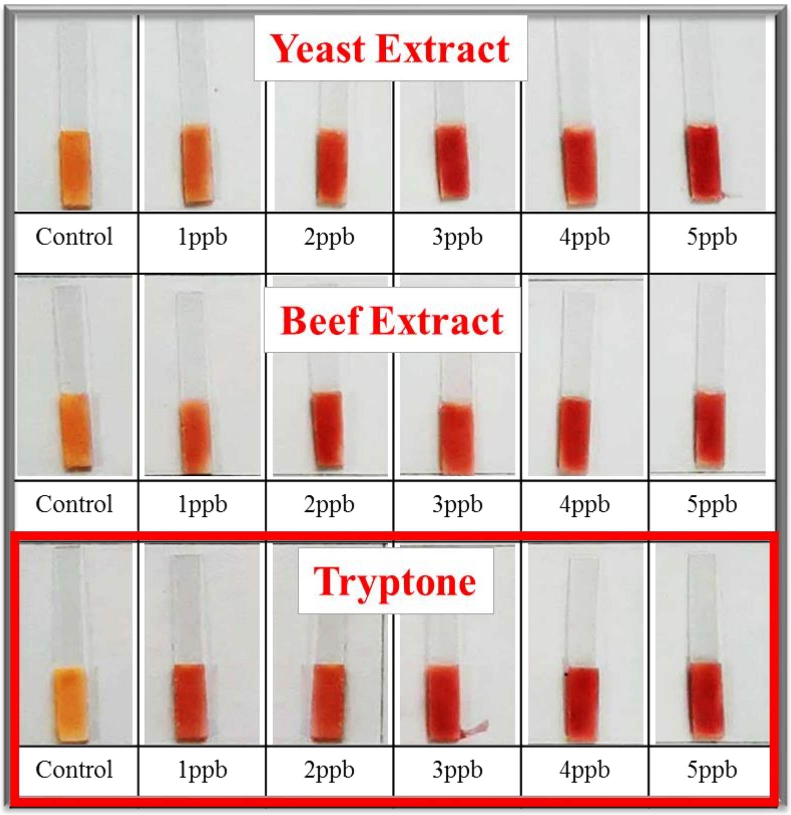

Supplementary Figure S12. Screening of nutrients at different concentration of amoxicillin

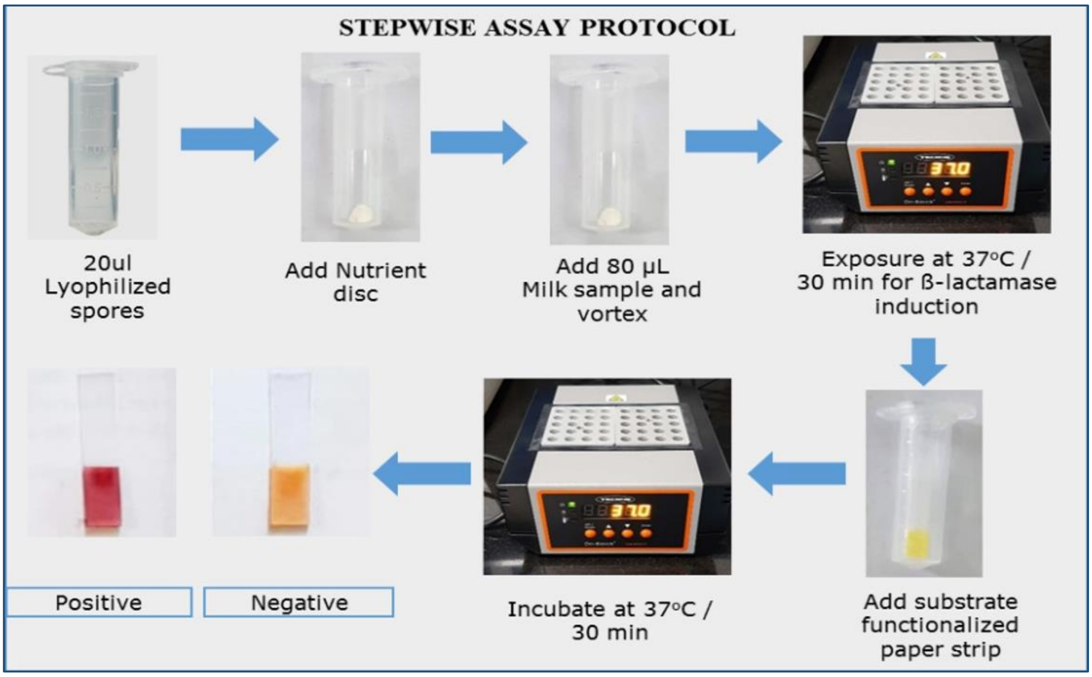

Supplementary Figure S13. Optimized assay protocol

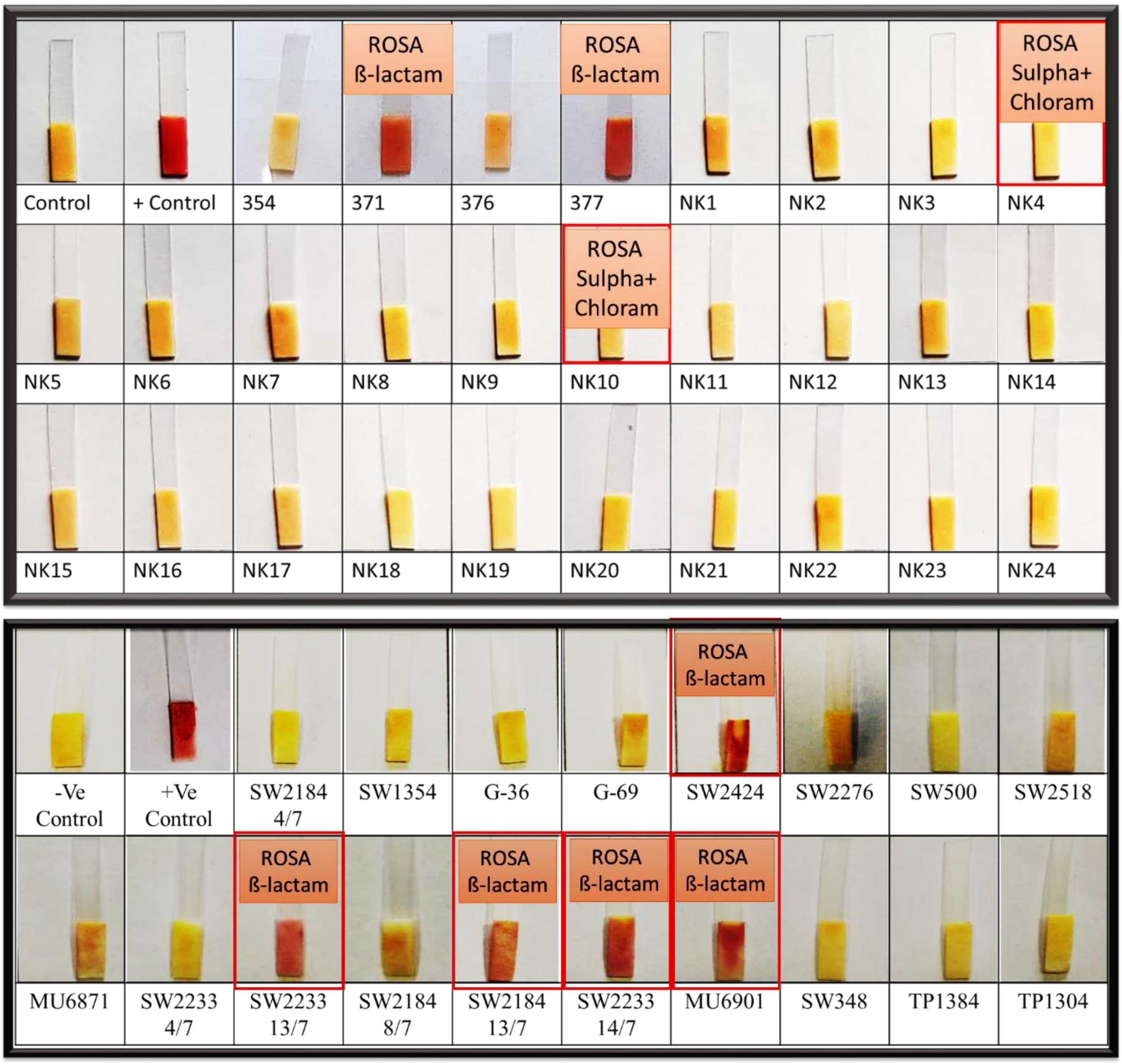

Supplementary Figure S14. Evaluation of Strip Based Test under Field Conditions with Raw Milk

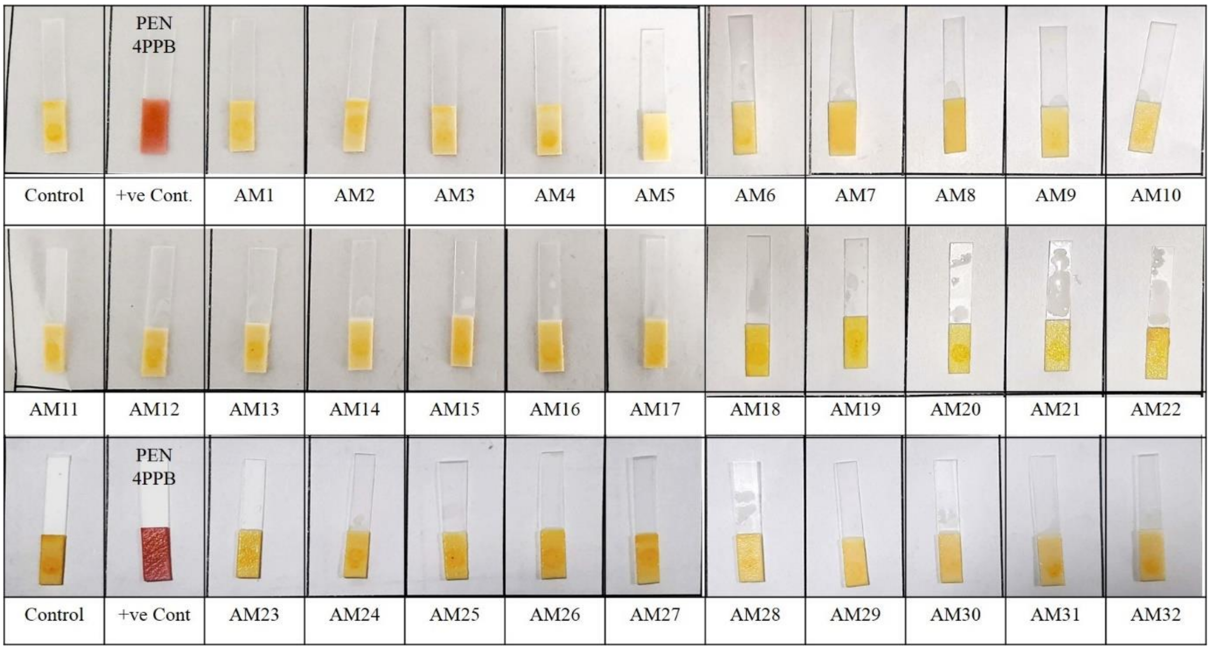
PEN
4PPB
Control
+ve Cont.
AM1
AM2
AM3
AM4
AM5
AM6
AM7
AM8
AM9
AM10
AM11
AM12
AM13
AM14
AM15
AM16
AM17
AM18
AM19
AM20
AM21
AM22
PEN
4PPB
Control
+ve Cont
AM23
AM24
AM25
AM26
AM27
AM28
AM29
AM30
AM31
AM32

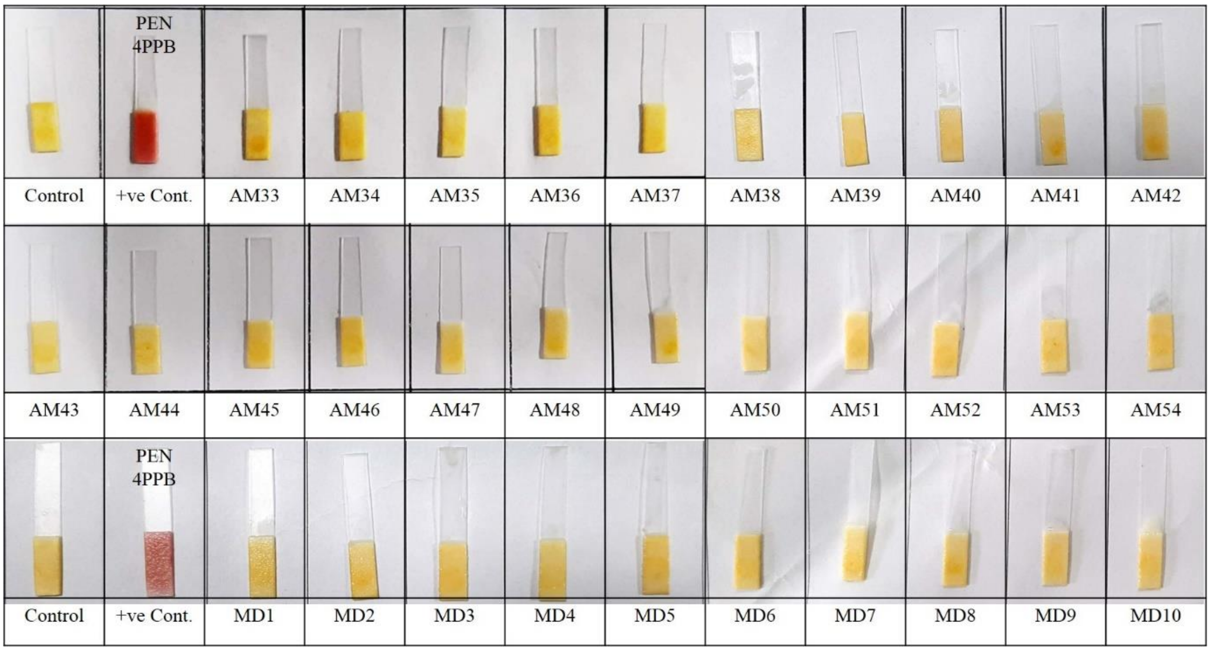
PEN
4PPB
Control
+ve Cont.
AM33
AM34
AM35
AM36
AM37
AM38
AM39
AM40
AM41
AM42
AM43
AM44
AM45
AM46
AM47
AM48
AM49
AM50
AM51
AM52
AM53
AM54
PEN
4PPB
Control
+ve Cont.
MD1
MD2
MD3
MD4
MD5
MD6
MD7
MD8
MD9
MD10

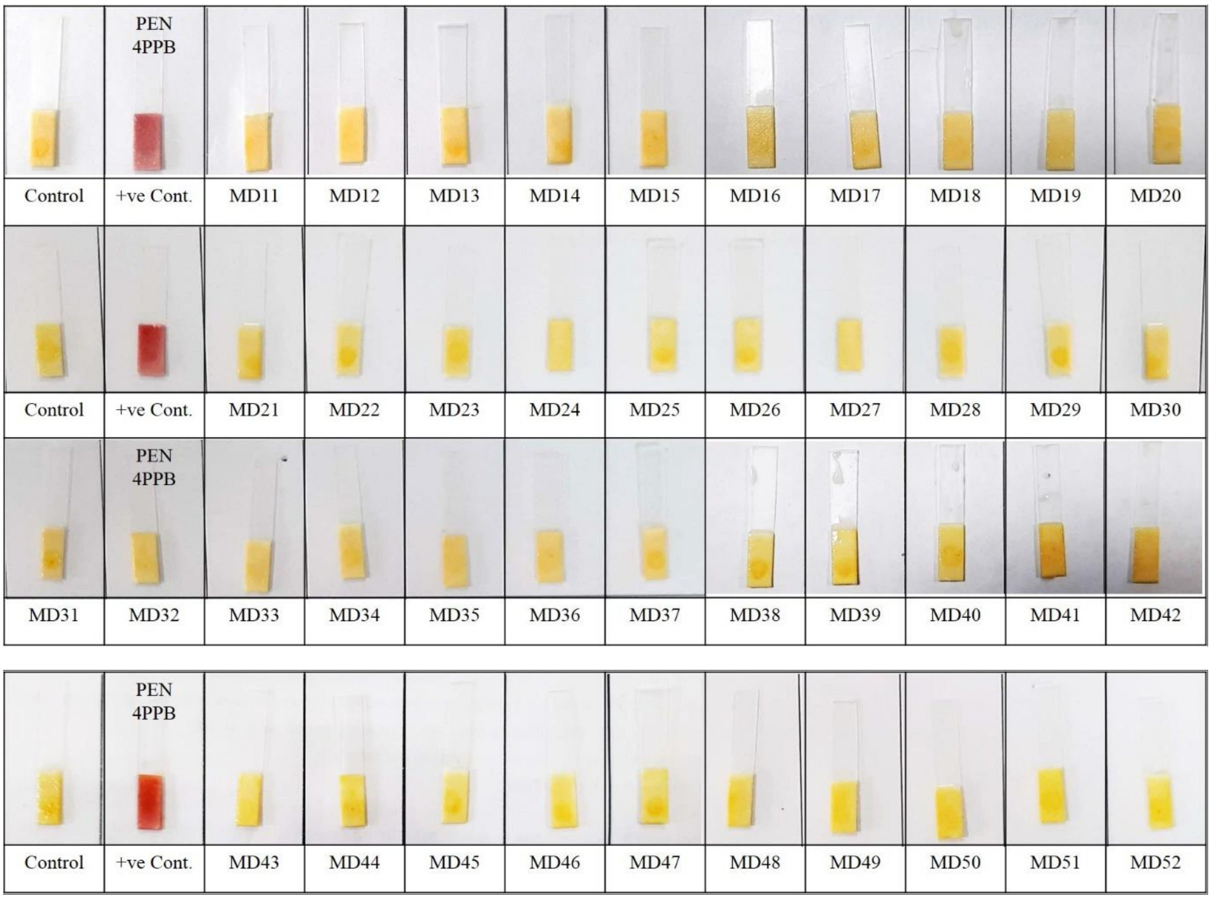

Supplementary Figure S15. Evaluation of the developed kit with pasteurized milk samples
